# Extend the Survival of Human Sperm In Vitro in Non-Freezing Conditions: Damage Mechanisms, Preservation Technologies, and Clinical Applications

**DOI:** 10.3390/cells11182845

**Published:** 2022-09-12

**Authors:** Qingyuan Cheng, Liman Li, Min Jiang, Bo Liu, Yang Xian, Shasha Liu, Xiao Liu, Wenrui Zhao, Fuping Li

**Affiliations:** 1 Department of Andrology, Sichuan Human Sperm Bank, West China Second University Hospital, Sichuan University, Chengdu 610066, China; 2 Key Laboratory of Birth Defects and Related Diseases of Women and Children of Ministry of Education, West China Second University Hospital, Sichuan University, Chengdu 610041, China

**Keywords:** human sperm, non-freezing preservation, in-vitro culture, semen pre-treatment, sperm injury, semen extender

## Abstract

Preservation of human spermatozoa in vitro at normothermia or hypothermia maintaining their functions and fertility for several days plays a significant role in reproductive biology and medicine. However, it is well known that human spermatozoa left in vitro deteriorate over time irreversibly as the consequence of various stresses such as the change of osmolarity, energy deficiency, and oxidative damage, leading to substantial limitations including the need for semen examinations, fertility preservation, and assisted reproductive technology. These problems may be addressed with the aid of non-freezing storage techniques. The main and most effective preservation strategies are the partial or total replacement of seminal plasma with culture medium, named as extenders, and temperature-induced metabolic restriction. Semen extenders consist of buffers, osmolytes, and antioxidants, etc. to protect spermatozoa against the above-mentioned adverse factors. Extended preservation of human spermatozoa in vitro has a negative effect on sperm parameters, whereas its effect on ART outcomes remains inconsistent. The storage duration, temperature, and pre-treatment of semen should be determined according to the aims of preservation. Advanced techniques such as nanotechnology and omics have been introduced and show great potential in the lifespan extension of human sperm. It is certain that more patients will benefit from it in the near future. This review provided an overview of the current knowledge and prospects of prolonged non-freezing storage of human sperm in vitro.

## 1. Introduction

Owing to the progress in male reproductive medicine and assisted reproductive technology (ART), there is a growing demand for preserving human spermatozoa in vitro, in most cases residing in the liquefied seminal plasma or culture medium. However, human spermatozoa left in vitro irreversibly deteriorate over time, beginning at approximately one hour after ejaculation as the result of many environmental stresses including increasing osmolarity, energy deficiency, bacterial growth, oxidative damage, and hypothermic injury [1,2,3,4,5,6]. Hence, for most semen examinations in the andrological laboratory, fresh semen samples are necessary. When sperm requires preservation for a longer time, from several hours to years, such as that required by fertility preservation of oligoasthenzoospermic or cancer patients, protective interventions must be performed to mitigate the premature aging of spermatozoa. For this purpose, the most predominant method by far is sperm cryopreservation. However, cryopreservation has its own limitations such as possible severe cryoinjury [7]. Furthermore, cryopreservation is not always the most optimal choice for extending the longevity of spermatozoa and is not even applicable to many clinical scenarios. For instance, sophisticated and expensive freezing equipment is indispensable for sperm cryopreservation, but its implementation is only available in a few central cities, which entails substantial inconvenience to patients who live in remote areas. Additionally, as of late, the delivery of semen examinations or ART procedures probably cannot be completed due to the quarantine caused by the COVID-19 pandemic [8,9]. In these cases, if sperm quality is able to be well maintained in the liquid state for a couple of days, patients can collect semen samples at home and ship them to an accredited laboratorial facility for extended semen examinations, ART, or fertility preservation. Some studies proposed that hypothermic preservation can be a substitute for cryopreservation if spermatozoa could be obtained surrounding the day of ART [10,11]. Besides, sperm acquired for ART undergo a prolonged incubation in artificial gamete or embryo cultural media, which may compromise its fertility [12,13]. Optimization of the sperm’s functional competence during this period is supposed to improve the clinical outcome of ART [14,15]. Therefore, prolonged storage of human spermatozoa in the liquid state play a critical role in the development of clinical screening and the diagnosis of male infertility, ART, in vitro cultural of germline cells, male fertility preservation, and other scientific and clinical uses.

Similar to the principle and approach of sperm cryopreservation, until now the basic strategies to prolong the lifespan of human spermatozoa in the liquid state in vitro are the partial or total replacement of seminal plasma with medium containing protectants, named as extenders, and temperature-induced metabolic restriction [16]. The attempt to perform liquid storage of semen in vitro can be traced back to the 1930s, initially in the field of artificial insemination (AI) [17] liquid storage of semen in vitro can be traced back to the 1930s, initially in the field of artificial insemination (AI) [18]. Bull and other domestic animals’ spermatozoa were chosen as the earliest research objects to meet the requirement of their reproduction and breeding. Phillips et al. reported that bull spermatozoa diluted with egg yolk-phosphate buffer at a temperature of about 10 °C could effectively maintain its motility for 150 h or more, as supported by pregnancy records [19]. Since then, this buffer has become the basic formulation of semen extenders and has been continuously revised. Small amounts of additives, such as sugars, gums, amino acids, and purified lipids are proven to be beneficial to sperm preservation [20]. In the 1970s, Tris with N-Tris (hydroxymethyl) methy-2-amino ethane sulfonic acid (TEST) plus egg yolk, called TEST-yolk buffer, which had been shown to be efficient in animals, was firstly introduced for the liquid storage of human semen, resulting in successful preservation for up to 72 h [21]. This finding provided the evidence that human sperm diluted with this buffer also could be maintained several days in vitro without freezing before ART. At the end of the last century, R.J Aitken et al. developed a diluent based on the TEST-yolk buffer for delayed laboratorial tests of human spermatozoa. With the aid of this, human semen samples are able to be preserved and transported for more than 24 h and still meet the requirements of performing a variety of semen examinations [22]. Moreover, using a similar buffer, human semen samples can be collected at home and successfully cryopreserved one day after delivery [23]. Except for the dilution of the TEST-yolk-based buffer, many artificial culture mediums designed for the purpose of ART, such as Ham’s F10 media and Human Tubal Fluid (HTF), are also used for the incubation of human spermatozoa over a longer period of time in research or clinical contexts [24]. A solution that simply contains glucose and Human Serum Albumin (HSA) was developed and human spermatozoa can wait for several weeks in it before IVF [10]. Nevertheless, the effect of preserving human sperm in the liquid state remains unsatisfactory and the liquid storage techniques need to be urgently improved. For example, many studies demonstrated that human sperm DNA fragmentation increased during prolonged incubation, whereas the viability could be maintained [5]. Besides, liquid storage of animal sperm, especially in livestock sperm, has constantly been a popular research topic for over half a century, with the continual rolling out of novel extenders, while the liquid storage of human sperm receives much less attention. Moreover, in contrast with cryopreservation technique of human sperm, existing studies focusing on liquid storage are limited and advances have lagged far behind regarding this field. However, there is no doubt that the development of liquid storage of human sperm in vitro helps to solve many key points of difficulties in andrological laboratorial contexts, ART, and male fertility preservation. Furthermore, this technique has great potential to improve developmental biology and reproductive medicine. Yet, the liquid storage techniques of human sperm have not been driven by these impending requirements.

To this end, we provide an overview of the current knowledge and state of liquid storage of human sperm in order to provide valuable information for the development of reproductive biology and medicine. Firstly, applications of liquid storage and shipment methods of liquid semen samples are discussed. Secondly, stresses imposed on spermatozoa residing in vitro are summarized. Thirdly, since the addition of extenders is the main strategy to preserve sperm in the liquid state, the components of extenders such as basic buffers, antioxidants, and osmoprotectants are described in depth. We also drew conclusions about the effects of prolonged preservation on sperm parameters and pregnancy outcomes and try to provide an optimal preservation strategy according to different clinical purposes and collection circumstances. Finally, a perspective is presented to give clues to further improve the effectiveness of non-freezing storage.

As previously noted, the prolonged liquid storage of spermatozoa has been used in animals for a long time. Regarding liquid storage techniques such as the TEST-yolk extender, we could find the use of it in animals prior to its use in humans. Many studies pertaining to new liquid storage techniques have been conducted in animal sperm until now, and these studies laid a solid foundation and paved the way for liquid storage of human sperm. Hence, we also discussed those which are recognized as significant and innovative. In addition, a large number of substances in liquid storage extenders, such as buffers, membrane protectants, and antioxidants, are also components of the sperm freezing medium. Their protective roles in liquid storage and cryopreservation are probably comparable. Therefore, many studies focus on the improvement and corresponding mechanism of these substances in cryopreservation are included in this review to elaborate their effects in the liquid storage. Besides, novel protective factors applied in cryopreservation are discussed concisely, which could shed lights on developing liquid storage extenders in the future.

## 2. Aims of Prolonged Non-Freezing Storage

### 2.1. Laboratorial Test

Prolonged non-freezing storage of human sperm has the potential to promote andrological laboratorial tests. Infertility affects approximately 15% of couples, in which male factors are responsible for approximately half of the cases [25]. The reasons for male infertility are various and advanced and comprehensive semen examinations are increasingly in demand. Semen samples differ from other bodily fluids such as blood or sputum, as they must be tested immediately, usually within 1 h, otherwise some of the essential sperm parameters will decline. It is reported that sperm motility is observed to decrease a few hours after ejaculation, at a rate of about 5% to 10% per hour [1]. These specificities of the semen samples require that anyone who needs sperm analysis must attend the testing facility. Although it is not difficult for patients that just need routine semen analysis, which could be tested in most departments of clinical laboratories, it is inconvenient and unfriendly for patients that need advanced semen assays, for instance, DNA fragmentation analysis, or have ejaculatory difficulties and mental anxiety masturbating outside. Besides, semen assessment in decentralized laboratories causes concerns regarding interlaboratory variations and quality control. The development of prolonged non-freezing storage technology helps to address the aforementioned problems. Studies state that all aspects of sperm function including viability, motility, and acrosome reactions, etc. can be effectively preserved by simply treating freshly produced semen samples with a diluent medium named as citrate-egg yolk buffer over a period of 24 h at room temperature, which allows patients to collect semen samples in other sites and then mail them to a centralized and accredited diagnostic laboratory when full evaluation of human sperm functions is available and high standardization eliminates the aforementioned interlaboratory variations [22,23]. Although it is very difficult to preserve semen samples effectively, especially for laboratory assessment, which requires that all the sperm parameters remain entirely unchanged, it is beneficial to many patients, in particular those who need semen analysis but for whom it is not convenient to attend andrological facilities, for example, those living in remote areas or who are disabled. Moreover, it can help patients to obtain more standardized sperm quality evaluation.

**Figure 1:**
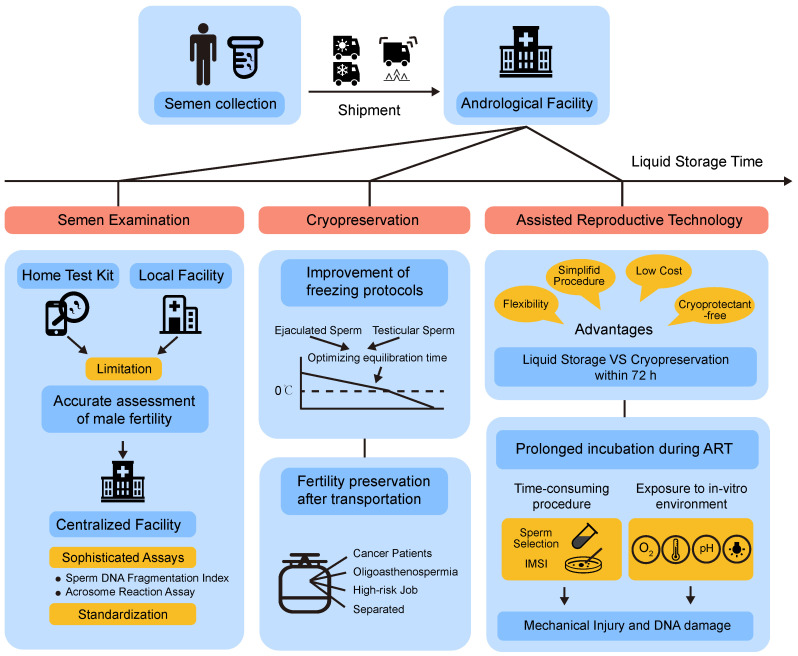
Scientific and clinical applications of prolonged liquid storage. Mode diagram summarizes the shipment and aims of prolonged storage of human spermatozoa in the liquid state.

Except for the application of non-freezing storage technology, home testing kits for semen analysis have been designed in recent years. Indeed, a variety of products that could analyze sperm concentration, viability, and motility are now commercially available, such as SpermCheck Fertility and Men’s Loupe [26]. Moreover, new assays, serving as adaptations and substitutions for the original, have been developed. For instance, some objective biochemical markers remain unchanged after overnight shipping, such as sperm creatine kinase (CK), which was identified and used for the evaluation of sperm maturity and function, estimated by conventional sperm parameters such as sperm motility and morphology. Therefore, the detection of these markers could also reflect the attributes of the initial semen well even better [27]. Another example is a new method for delayed assessment of sperm motility. This method detected sperm mitochondrial activity through fluorescent dye, and the emission levels showed close correlations with sperm motility at the time of the ejaculation [28]. In addition, it was found that air-dried semen could be stored at −22 °C for up to one month before DNA fragmentation testing [29]. However, semen analysis using commercial products at home has many limitations such as false-negative results and inadequate quality control. Moreover, home semen test methods can measure basic sperm parameters but are unable to complete advanced tests, so they cannot replace laboratory tests. New assays that try to overcome the delay of transportation also have the potential to be alternatives, though few are used in laboratory tests and their clinical use needs to be further evaluated. Compared with semen home test kits or new assays, short-term liquid storage is considered as the best method for delayed laboratory testing of human semen (Figure 1).

### 2.2. Assisted Reproductive Technology (ART)

Liquid storage of sperm presents several advantages that are particularly useful in ART. When spermatozoa are just needed to stay in vitro within a certain period, usually no more than 72 h, compared with cryopreservation, storing semen in the liquid state could simplify harvesting, preservation, and retrieval procedures, save money, and avoid the use of cytotoxic protectants (Figure 1). In practice, it could be applied for artificial insemination (AI), which is often performed following the semen stored for a period time in vitro. In the case of animal breeding such as stallions and boars, the transportation or short-term storage of their semen before AI is extreme important for their genetic requirements. Most of these processes could be completed within 72 h, and thus liquid storage of spermatozoa has been widely used in AI of livestock for a long time [30]. During the period of in vitro storage, they need liquid storage techniques to preserve sperm against rapid deterioration and spoilage, which also exhibits the potential for clinical use in humans. On the basis of animal applications, studies showed that human spermatozoa recovered after refrigeration in TEST-Yolk buffer show better viability and fertilizing capacity following short-term storage between 24 and 96 h [31,32,33]. A method using electrolyte-free solution to preserve semen samples for delayed ART up to 2 weeks was also invented [34]. Interestingly, some studies showed that short-term liquid storage has the potential to be a sperm selection method in ART [31,33,35]. In this case, spermatozoa with better quality characteristics and superior fertilizing capacities could be yielded after a short-term cryostorage period in TEST-Yolk buffer. The decrease rate of sperm motility after different hours of incubation can provide support in the clinical decision-making of the choice of In Vitro Fertilization (IVF) or Intracytoplasmic Sperm Injection (ICSI) [36]. Recently, it has been proven that home semen collection within 2 h of processing for IVF/ICSI procedures has no negative effect on sperm parameters or early IVF/ICSI outcomes in retrospective studies [8,9]. This suggests that collecting semen sample at home has the potential to be a safe strategy to protect patients from coronavirus disease 2019 (COVID-19) pandemic. Thus, for men who may be unable to provide sperm due to separation, ejaculatory difficulty, or oligoasthenzoospermia, they can preserve their semen in a liquid state several days before assisted fertilization, avoiding sperm injuries after freezing and simplifying sperm handling procedures, which provide more flexibility for timing the ART [37]. Prolonged non-freezing storage of human sperm may also bring about changes in sperm donation. Due to semen testing being one of the only steps during the screening process, men who plan to donate sperm are required to attend the sperm bank to complete all screening steps such as physical exams and genetic tests. Nevertheless, men qualifying as sperm donors may send semen samples instead of making several appointments in the bank to provide enough semen samples, taking advantage of the non-freezing storage technique. This may attract sperm donors from remote places. Moreover, a “donation kit” which contains TEST-yolk buffer in vials has been used to prepare and pack and then ship a sperm donation to a recipient in another city since “direct donations” that sperm donations made to a known recipient have emerged in some countries (https://www.knowndonorregistry.com/ accessed on 10 September 2022).

Prolonged in vitro liquid storage of spermatozoa is also a necessary part of IVF manipulation. Sperm are subject to many laboratory interventions during the IVF procedure. At first, spermatozoa with higher motility and better morphology need to be selected from the whole semen. The most-used sperm selection techniques are the swim-up method and centrifugation through discontinuous density gradients of silane-coated silica colloidal particles (DGC). Both methods are time-consuming. For example, it takes several hours to perform the direct swim-up process [38]. Studies also reported that these sperm preparation methods may give rise to the evaluated DNA fragmentation [39]. Some advanced sperm selection techniques developed in recent years, such as magnetic cell sorting (MACS) with annexin V conjugated beads and intracytoplasmic morphologically selected sperm injection (IMSI), require longer cultural or handling time, increasing the risk of oxidative stress and consequent DNA damage. ICSI, especially IMSI procedures, are more likely to take extra time. Secondly, spermatozoa may be incubated for a long time while waiting for the insemination procedure because insemination and ovulation are often not synchronized [17]. Whole in vitro manipulations may last several hours, exposing sperm to many exogenous physical and chemical factors. These are significantly different from the natural conception process, including change of temperature, pH, O_2_ tension, centrifugation, exposure to visible light under the microscope, and, apparently, prolonged storage in in vitro cultural media. For example, it is reported that prolonged sperm manipulations during assisted reproduction therapy should be performed at 21 °C rather than 37 °C in order to avoid decreasing the morphologic integrity of the sperm nuclei [40]. All of the manipulations involved in sperm handling, washing, selection, and ICSI processes may induce many adverse effects and impair ART outcomes, such as mechanical injury and excessive production of ROS. Oxidative stress (OS) is of great concern due to its harmful effects on sperm functions that play a role in fertilization and, furthermore, damage to DNA; the direct association between sperm OS and ART outcomes after IVF is still a matter of debate [25]. Except for ejaculated spermatozoa, testicular spermatozoa yielded from testicular sperm extraction (TESE), fresh or frozen-thawed, are also subjected to in vitro culture before ICSI [14]. Testicular spermatozoa are more fragile in comparison with ejaculated spermatozoa and higher requirements have been put forward for maintenance and activation of testicular spermatozoa. Prolonged storage of sperm also paves the way for the in vitro culturing of other germline cells such as spermatogonia stem cells [41,42].

### 2.3. Cryopreservation

Liquid storage of human sperm provides flexibility not only for ART, but also for semen cryopreservation. In recent years, the demand for fertility preservation in men for future ART, oncologic care, and personal reasons has increased dramatically [43] (Figure 1). Cancer patients are highly advised to preserve their sperm before accepting chemotherapy or radiotherapy, which can render them permanently infertile [44]. In addition, for men engaging in high-risk jobs or even the normal male population, “fertility insurance” is also in demand. To date, cryopreservation is the only method for long-term storage of human spermatozoa. However, semen needs to be frozen immediately after ejaculation to prevent deterioration. Meanwhile, the freezing process requires specialized ultra-low-temperature equipment, and the frozen semen sample must be stored at accredited sperm banking facilities. Thus, men who need fertility preservation must come to the facility for semen collection, which is only available in a few large cities. In China, there are only 23 human sperm banks in total. Strict conditions severely limit their accessibility to those from remote locations or those suffering from mobility disabilities, constituting a challenge for successful fertility preservation care. The development of liquid storage technology allows for more flexible timing and sites of semen procurement, for example, collecting semen at home and delivering it to the facility for cryopreservation. Within this period of time, semen samples could maintain their fertility potential and obtain the comparable post-thawing efficiency after delayed cryopreservation, thus strongly facilitating the accessibility of male fertility preservation care [45].

The development of liquid storage technology also benefits sperm cryopreservation protocols (Figure 1). Since the efficiency of conventional freezing method and vitrification remains inconclusive, the conventional slow-freezing method still acts as the main approach used for human semen cryopreservation [46,47]. The cooling process from normothermia to about 5 °C and the following equilibration time in refrigeration conditions is identified as a crucial step affecting cryopreservation outcomes in the conventional freezing procedure. Cooling alters biomolecules and biological processes in sperm cells such as ATP depletion, lipid damage, and oxidative stress. These changes might compromise sperm function and even be sublethal, thus shortening the post-thaw sperm survival [7]. Typically, sperm plasma membrane is subject to phase transitions about 4–10 °C, which makes it become fragile and lose membrane integrity [48]. It has been reported that equilibration with a TEST-yolk-catalase-based extender for about 24 h in the refrigerator before freezing helps to enhance the freezability of sperm and improve the post-thaw sperm qualities via protecting cell membranes from cholesterol efflux [49]. It suggested that optimizing the cooling and equilibration phase that occurs in the liquid state plays a role in the outcomes of the whole freezing procedure. Recently, the metabolomic signature of spermatozoa constructed during the equilibration time at 17 °C for 24 h was found to play a role in the sperm cryotolerance. Many metabolites such as inosine, hypoxanthine, and creatine might be critical additives in the liquid storage medium. Meanwhile, the up-regulation of metabolic pathways including arginine and proline suggests that targeting these pathways has the potential to improve the efficiency of liquid storage [50].

### 2.4. Shipment of Semen Samples

For men who live far from fertility facilities and require laboratory testing or fertility preservation, sperm liquid storage technology makes the transportation of their fresh semen samples from a distant site possible. In order to ship them successfully, a liquid preservation solution that can maintain sperm parameters and fertility potential is insufficient. It is important to develop a strong and sealed container that is able to withstand the issues in transit, such as preventing the semen sample from evaporation or pouring down. It is also important to maintain the stability of the environment outside, especially controlling the temperature. Semen samples may be shipped from tropical regions to temperate regions, or in different seasons. Thus, the temperature change can be drastic during the shipment, which impacts the period of time in which the sperm quality can be maintained. To overcome these problems, a collection device called Bio-Tranz^TM^ system for cryo-stored and transport human semen at 5 °C was developed [51]. The specimens were diluted with TEST yolk buffer and stored for 24 h. Although sperm motility and membrane integrity were significantly decreased following storage, the pregnancy rates remained unaffected. Subsequently, researchers from Andrology Center of the Cleveland Clinic designed and standardized a sperm collection and transport kit named “NextGen”, which is a first-of-its kind product that allows patients to collect the semen sample at home and package it for overnight mail delivery to Cleveland Clinic’s Andrology Laboratory for cryopreservation and long-term storage. This kit includes a transport media, a semen specimen container, and a cooling sleeve [45]. The post-thaw sperm motility, total motile sperm, and percent cryosurvival rate were similar between the control group of onsite collection and offsite collection via shipment using NextGen kit [24]. An adequate number of spermatozoa meeting the requirements of ART were available, which showed the feasibility of this new product in male fertility preservation. The decrease of sperm quality was found, suggesting that it is not suitable for use in laboratory testing. Recently, glass-forming water-salt systems for hypothermic preservation of semen were developed, which retained higher sperm viability and motility, suggesting a promising approach in this field [52]. Nevertheless, further research is needed to investigate the effect of this kit in patients with oligoasthenospermia, similar to studies reported by R.J Aitken [22]. In summary, developing a “package” with the optimization of preservation solution and temperature control could prolong the transport time of semen samples, expanding the fertility preservation options for men throughout the country (Figure 1).

## 3. Sperm Injuries during Prolonged Non-Freezing Storage

**Figure 2:**
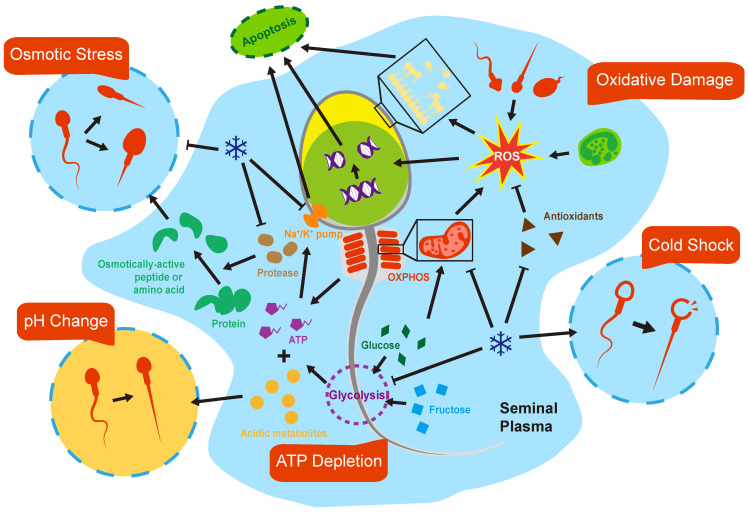
Biological mechanism of sperm damages in vitro. Spermatozoa are subjected to a variety of stresses once exposed to in vitro environments in the liquid state, which compromise their lifespan.

Sperm is one kind of cell, and there are many common injuries for sperm and other cells that need in vitro preservation for a longer period of time. One of the main problems of somatic cell preservation is the loss of Extracellular Matrix (ECM), which probably becomes a problem for the preservation of testicular tissues but not for sperm [53]. These injuries accumulate, which in turn manifest as a decline of sperm motility, membrane and DNA damage, and, ultimately, cellular apoptosis and necrosis. Sperm freezing and thawing focus on cryoinjuries, and the impact of the metabolism should be highlighted in liquid storage (Figure 2).

### 3.1. pH and Osmolarity

The supply and maintenance of optimum culture environments is fundamental for the longevity of sperm during preservation in the liquid state. Two environmental stress factors that have been identified as being of great importance are pH and osmolarity. Spermatozoa might be subjected to the pH and osmolarity that significantly deviate from the physiological environment once being expelled from the body, which is detrimental to its post-ejaculated life. The pH and osmolarity of semen depend highly on two factors: its inherent individual characteristics and the length of time since ejaculation. Compared with oocytes and embryos, sperm cells have little capacity to maintain the stability of intracellular pH [54,55]. In contrast, semen possesses nearly the highest buffer capacity among other bodily fluids, mainly derived from HCO_3_/CO_2_, citrate, and protein in seminal plasma [56]. However, there is a limit of such an ability. The pH of normal fresh ejaculate is near neutral, ranging from 7.2 to 8.2. Then, it increases shortly due to the depletion of CO_2_ in vitro and further decreases gradually when exposed for a longer time as the result of accumulation of acidic metabolites. It is generally accepted that acidic environments are toxic to spermatozoa, rendering sperm immobilized [57]. Besides, lower pH could occur in diseases such as retrograde ejaculation or seminal vesicles obstruction [3]. These types of abnormal seminal plasma are apparently an unqualified media for spermatozoa at the beginning [58]. Interestingly, extracellular alkalization can reinitiate sperm motility after staying in low-pH conditions for a while or long-term cold preservation in the electrolyte-free solution [3,59]. Therefore, it is imperative to introduce buffer substances aiding the stabilization of pH during the whole liquid storage period, which is critical for preserving sperm viability and fertilizing competence.

As with pH, osmolarity in human semen varies in individuals and depends on factors inherent to each semen sample [60]. It is reported that normal fresh ejaculate after liquefaction showed remarkable hyperosmolarity compared with blood plasma, being approximately 320–360 mOsm/kg [3,56,61]. However, a much higher or lower semen osmolarity can be observed in asthenozoospermia, suggesting its role in pathogenesis of male infertility [62,63,64]. Notably, along with semen aging, semen osmolarity increases continuously, reaching approximately 500 mOs/kg after 24 h post-ejaculation [6,63,64]. The osmolarity of semen is mainly determined by sugars, proteins, small organic molecules, and ionic salt concentrations in seminal plasma [56]. The increase in semen osmolarity during liquid storage is influenced by a variety of factors including enzymatic activities and prostatic secretion [65]. Reasonably, abnormal osmolality of human semen would result in changes of sperm cell volume while residing in the ejaculate, bringing risks of osmotic injury. However, spermatozoa go through a significant variation of osmolarity during sperm maturation and intercourse, from about 290 mOsm/kg close to serum in testis, rising to above 400 mOsm/kg in the epididymal and vas deferens, and then reversal to 290 mOsm/kg in female tract fluids after ejaculation [66,67,68]. These changes of osmolarity, physiologically, are enough to cause swelling or shrinkage of cells, compromising their function. In order to counteract that, spermatozoa own the ability to regulate their volume effectively via the efflux or influx of osmolytes presented in cell or seminal plasma to drive water movement that adjusts the cell volume. Nevertheless, spermatozoa are vulnerable to osmotic stress in vitro and both too high and too low semen osmolarity are detrimental to sperm parameters [69]. Among these, sperm motility and kinetics parameters are especially sensitive to the change of osmolarity because, for instance, hypoosmolality firstly causes coiling or angulation of the flagellum and slight swelling could slow forward progression [64,66]. Notably, sperm, once adapted to high osmolarity when left in the liquefied ejaculate, are more likely to decline if contacted with a medium with a lower osmolality, which usually occurs in the ART procedure [70]. Moreover, continually rising osmolarity of seminal plasma after prolonged storage may be so strong that it is beyond the rectification of volume regulation mechanisms, resulting in more severe damage. Considering the possible key influencing role of osmolarity, scientists have developed many methods to help stabilize the osmolarity in the prolonged storage, such as lowering the preservation temperature, the addition of inorganic or organic osmolytes, and enzymatic inhibitors. However, early dilution of semen with isotonic buffers is so far considered as the most effective and convenient way to stabilize osmolarity during liquid storage [65].

### 3.2. Metabolism and ATP Depletion

Sperm motility, hyperactivation, and capacitation, which are essential for successful fertilization, are highly energy-dependent processes. As with somatic cells, the predominant metabolic pathways that spermatozoa use to produce adenosine triphosphate (ATP) are glycolysis and oxidative phosphorylation (OXPHOS). Despite OXPHOS being much a more efficient metabolic pathway for ATP synthesis, glycolysis has a dominant place in the tail region of spermatozoa due to ATP produced by mitochondria not being able to diffuse sufficiently throughout the remote end of the flagellum [71]. However, the preferred metabolic pathway used to generate energy chosen by spermatozoa is highly species specific. Although remaining inconclusive, it is reported that spermatozoa of livestock animals, such as bovines and stallions, rely on OXPHOS for almost all physical procedures. Human spermatozoa, as well as rodents, depend predominantly on glycolysis to generate ATP for supporting motility [72]. Nevertheless, OXPHOS are required to provide ATP for hyperactivation, acrosome reaction, and capacitation in human spermatozoa. In addition, mitochondria play an important role in sperm maturation and differentiation [73]. Mitochondrial dysfunction in human spermatozoa has been shown to be associated with deprived energy status, oxidative stress damage, and premature apoptosis [74].

During prolonged liquid storage of human semen, a perquisite for maintaining sperm quality is adequate energy supply. The main energy sources of sperm are glucose, fructose, and pyruvate acids in semen [75]. Despite glucose concentration measured in semen (0.3 μmol/mL) being far lower than fructose (12.4 μmol/mL), spermatozoa prefer to utilize glucose, which accounts for nearly half of sugar consumption [76]. The sugars stored in semen are enough to make spermatozoa maintain high motility for serval hours, though they will gradually be depleted during prolonged storage. Energy deficiency leads to dysfunction of some essential cellular processes such as the Na^+^/K^+^ ATPase. At the same time, metabolites are constantly released into seminal plasma. Studies using ^13^C-magnetic resonance spectroscopy to track sperm metabolism observed a significant accumulation of lactate and bicarbonate derived from glucose, fructose and pyruvate over 48 h post-ejaculation, which indicates the rapid glycolysis and OXPHOS occurring in human semen [77,78]. However, sperm cells and semen substances do not have enough capacity to neutralize or eliminate them. When these metabolites accumulate to a certain degree, they will have a detrimental effect on sperm quality. For example, they could have implications for the pH and osmolality of semen, in which sperm parameters such as motility are sensitive to these changes. Among these by-products, reactive oxygen species (ROS) are of great concern. The chief source of ROS in semen is OXPHOS in the mitochondria of spermatozoa. Glycolysis contributes to significantly less ROS than OXPHOS. Mild ROS serve essential functions in capacitation, acrosome reactions, and sperm hypermotility. However, excessive ROS will induce oxidative stress, which plays a role in male infertility, and is considered as an important adverse factor in liquid storage and cryopreservation of sperm [79,80].

### 3.3. Oxidative Stress

Higher oxidative stress and lower antioxidant capacity were found in the semen of about 25% of infertile men [81,82]. Oxidative stress is also considered as an intractable problem during the prolonged in vitro liquid storage of semen. We speak of oxidative stress when production of oxidative substances overwhelms antioxidants capacity. Reactive oxygen species (ROS) are major cellular oxidative substances, including free radicals and non-free radicals such as superoxide anion (O_2_-), hydroxyl radical (-OH) and hydrogen peroxide (H_2_O_2_). These ROSs lead to oxidative damage to proteins, nucleic acids, lipids and other biomolecules in cells and tissues.

ROS in the semen mainly originate from endogenous sources. One of the main sources are cells presented in seminal fluids, including immature spermatozoa, morphologically abnormal spermatozoa, and leukocytes, the latter producing 1000 times more ROS than spermatozoa [83]. Moreover, dead spermatozoa are also considered as an important source of ROS, which could increase significantly during sperm storage and manipulation [84]. However, as mentioned above, the other main ROS producers are sperm metabolism, especially oxidative phosphorylation reaction (OXPHOS) in the mitochondria of spermatozoa [79,85].

Unfortunately, spermatozoa are some of the most vulnerable cells to the ROS. Firstly, the plasma membranes of spermatozoa have an abundance in polyunsaturated fatty acids (PUFA), which is particularly susceptible to ROS. Oxidative stress leads to the lipid peroxidation of sperm plasma membrane, rendering the sperm inactive, immotile and loss of membrane integrity. Secondly, ROS attacks the nuclear and mitochondrial DNA of spermatozoa, especially DNA with defective chromatin compaction, and the DNA damage detection and repair ability of spermatozoa is fragile. The DNA damage caused by oxidative stress in spermatozoa includes strands breaks and DNA fragmentation, which impedes essential sperm fertile function and induces cell apoptosis. In addition, the main cytoplasm of spermatozoa is removed during maturation, causing a lack of intracellular antioxidant enzymes. Therefore, the elimination of oxidative substances is highly dependent on antioxidants exists in seminal fluid [86]. Finding states that seminal plasma is one of the largest sources of some essential antioxidants such as SOD [87]. Although ROS production has been involved in the pathophysiology of male infertility, actually, ROS plays a dual role in sperm. It was found that mild oxidative stress in sperm supports many essential physical activities, such as capacitation, acrosome reaction, and spermatozoon–oocyte fusion. In a normal state, there is a balance between the generation of ROS and the capacity of antioxidant defense systems in the fresh ejaculates and could be maintained for several hours. However, along with the prolongation of storage time, sperm cells and leukocytes in semen release ROS constantly. At the same time, the dead sperm rate increases, acting as another important contributor to ROS. Along with the constant accumulation of ROS, limited antioxidants deplete rapidly without supplementation. This balance between production of ROS and the antioxidant capacity will be broken inevitably. The lipid peroxidation, DNA damage, and apoptosis occur as a result of excessive oxidative stress, taking part in the deterioration of sperm quality parameters during the prolonged storage of semen.

Given that the chief source of ROS is sperm metabolism, a simple approach to assuage oxidative stress is to inhibit metabolism through hypothermic preservation. However, hypothermia could also compromise the activity of antioxidant enzymes. The cooling condition is a double-edged sword, because the benefits of ROS reduction may not counteract the detrimental effects of cooling itself, for instance, the dysfunction of the Na^+^/K^+^ pump, promotion of cholesterol efflux, and weakness of the sperm membrane integrity [84]. The influence of temperature on sperm motility and ROS was evaluated and the results showed that semen samples should be stored at 37 °C after collection and during transportation and processing within one hour [88]. Antioxidant supplementation in extenders is believed to be the main solution to alleviate the detrimental effects of the oxidative stress during the liquid storage of sperm. Therefore, a variety of antioxidants have been tested. Antioxidants presented in spermatozoa or seminal fluids, such as SOD and CAT, amino acids such as GSH, taurine, and proline, and exogenous antioxidants extracted from plants such as resveratrol and curcumin have been used in preservation of animal and human semen. However, their effects remain controversial.

Due to the heterogeneity of semen quality among males, it is hard to achieve a consistent protection outcome of each semen sample after the standard strategy of antioxidant addition. Semen samples with abnormal qualities, for instance, those of oligoasthenzoospermia patients, are more susceptible to oxidative stress, causing a negative influence on the subsequent ART procedure [25]. In addition, it is supposed that semen samples with higher levels of oxidative stress or weak antioxidant capacity may also be subjected to faster deterioration of sperm parameters during prolonged storage [89,90,91]. Considering that various unhealthy lifestyle-related factors and external environmental factors all could impede male infertility, the adoption of lifestyle and antioxidant therapy could be helpful for the reduction of ROS production and the improvement of antioxidant capacity [82,83]. For example, it is reasonable to recommend that oligoasthenzoospermia patients receive treatment before the preservation of their semen.

### 3.4. Cold Shock

Except for energy supplements and antioxidant addition, another approach avoiding the deleterious effect of the metabolite accumulation and subsequent oxidation activation is the decrease of temperature through reduction of metabolism. Controlling the temperature above the 0 °C, mostly within 2–5 °C, known as the “refrigeration condition”, could be a traditional but effective method. However, the refrigeration procedure of sperm towards this temperature would face the risk of “cold shock”. When rapidly cooling to the near-freezing point of water, sperm may be cold-shocked, which presents as the irreversible depression of motility and metabolic activity, and disruption of acrosome and plasma membrane [92]. The susceptibility of cold shock varies among mammalian sperms. For instance, bull, pig and stallion spermatozoa are highly susceptible to cold shock, while rabbit and human spermatozoa are less sensitive [93]. The cold shock was soon proved to be the consequence of the thermotropic phase transitions in sperm cell membrane lipids [94]. When cooling rates are greater than a few degrees per minute, the sperm cell membrane is subjected to a transition from liquid-crystalline to gel phase. In this process, the flagellar activity decreases and solutes across cell membranes such as potassium and calcium leak, causing damage to sperm motility and intracellular organelles, and this phase separation happens abruptly [92]. It was found that the susceptibility of sperm to cold shock is highly correlated with the lipid composition of sperm membranes. The sperm membranes of pig and bull have a high ratio of polyunsaturated saturated fatty acids of the spermatozoan phospholipids and low levels of cholesterol, while the sperm produced by rabbit and human have a low ratio (about 1) and double the amount of cholesterol. Higher levels of saturated fatty acids enhance the cohesion, rigidity, and impermeability of the membrane structure, helping to maintain the membrane integrity during cooling [95]. In order to reduce the cold shock, one direct way is to identify the temperature range of cold shock sensitivity and control the cooling rate [96,97]. The other way is the addition of membrane protectants, such as egg yolk and soybean lecithin, which have shown great beneficial effects in refrigeration conversation of livestock sperm and more chemical defined membrane protectants have been identified in recent years [98,99,100,101]. Because of the high resistance to cold shock, this topic received less attention in cooled storage of human sperm [95]. However, using membrane protectant also improves the freezability of human sperm, so that they are widely applied in cryoprotective agents as essential ingredients. These are supposed to exert more important roles in the prolonged liquid storage of human sperm.

## 4. Effects of Extended Non-Freezing Storage of Human Spermatozoa on Semen Examination and Pregnancy Outcomes

To date, the influences of prolonged sperm preservation in the liquid state are mainly assessed by semen examinations or the change of sperm cells, resulting in a negative impact on the sperm parameters [102]. Nevertheless, the decline of sperm parameters during extended incubation may have no influence on fertilization rate [103]. The effects of it on pregnancy outcomes remain inconsistent. Therefore, on the basis of semen examinations, ART outcomes should be shown more attention in the evaluation of efficiency of extended preservation.

### 4.1. Sperm Parameters

Four sperm parameters, motility, oxidative stress, membrane integrity and DNA damage, receive the most attention (Table 1). Sperm motility is definitely a fundamental property of the spermatozoon and the decrease of it is significantly associated with male infertility [36]. Motile spermatozoa decrease progressively, from one-hour post-ejaculation, at a rate of approximately 5–10% per hour. The percentage of maximum motile sperm can be maintained for 4 h in most ejaculates and then decreases gradually [1]. Factors responsible for sperm motility are complex, including the calcium pathway, the cAMP-dependent protein kinase pathway, the activity of kinases and phosphatases, the ROS generation, and the changes of pH and osmolarity [104]. All these factors may be disturbed during liquid storage, leading to the decline of sperm motility much earlier than other routine parameters such as viability. Thus, the decline of sperm motility is believed to be a simple, sensitive, and reliable means to evaluate the effect of liquid storage and it is the most common parameter used in human spermatozoa. For example, when investigating the effect of storage duration and temperature, motility is the primary option from the 1970s to the present [105,106,107,108]. Except for total motility and progressive motility, a variety of motion parameters have been used for evaluating the prolonged non-freezing storage of human sperm with the development of Computer-assisted Semen Analysis (CASA). Addition of CYB significantly sustained all aspects of sperm motion parameters over 24 h including Curvilinear Velocity (VCL) and Average Path Velocity (VAP) [22]. It is found that penicillamine, as a thiol-containing compound, not only exerted a highly significant stimulatory effect on both total motility and progressive motility, but also generated a significant increase in VSL (Straight-line Velocity), VCL and VAP [109]. Furthermore, changes of motion parameters during the prolonged non-freezing storage are regarded as a valuable auxiliary indicator because they reflect the tail damage and volume regulation ability, especially caused by abnormal osmolality [64,70].

Oxidative stress, which has been identified as playing a vital role in the deterioration of post-ejaculated semen, as described in the previous section, is also a frequently used function parameter indicative of preservation efficiency. Notably, among the deleterious pleiotropic impacts arising from oxidative stress, sperm motility appears to be particularly vulnerable [129]. Nevertheless, the decrease of motile sperm has been the consequence of earlier oxidative injuries such as mitochondrial ROS generation [109]. Therefore, it is necessary to measure the oxidative stress directly and parameters more related to oxidative damage. The methods for directly measuring ROS level include chemiluminescence assays, static oxidation-reduction potential, and fluorescent probes such as dichlorodihydrofluorescein (H_2_DCFDA) [82]. Moreover, the total antioxidant capacity (TAC) in seminal plasma and the total ROS level also are important indicators reflecting the change of oxidative stress levels during the liquid storage of livestock semen [89,130,131]. Another important oxidative damage of sperm triggered by ROS is lipid peroxidation. It can be measured by the probe named as BODIPY C_11_[74]. Furthermore, 4-Hydroxynonenal (4HNE), as one of the end products of lipid peroxidation, has the potential to be a promising biomarker of oxidative damage. The production of 4HNE causes mitochondrial injury, and eventually results in DNA damage and apoptosis. The detection methods of 4HNE include flow cytometry, ELISA kit, mass spectrometry, and proteomic approach [132,133,134,135]. Since sperm mitochondria is an important source of ROS and the organelle that is susceptible to it, measurement of mitochondrial ROS production and mitochondrial damage is widely used for sperm quality evaluation during liquid storage [85,136]. For mitochondrial dysfunction, mitochondrial membrane potential (MMP), which reflects early mitochondrial injuries, is the most commonly used parameter. It can be detected using fluorescent probe JC-1 by flow cytometry. MMP is believed as one of the best parameters reflecting sperm mitochondrial function and mitochondrial energy status. Interestingly, it is reported that sperm with worse mitochondrial function, assessed by MMP, show a substantial decrease in sperm motility since the time of ejaculation. This finding suggested that some of the sperm samples may not suitable for prolonged storage [123]. It should be noted that the essential role of sperm mitochondria, not only affected by ROS, has been highlighted repeatedly so that MMP is widely evaluated in sperm with time after ejaculation [120,137,138]. Except for mitochondrial membrane injuries, other membrane damages including plasma membrane and acrosome integrity that may occur during liquid semen storage should be highlighted, since they are regarded as essential indicators of sperm injury. These damages are detected by a variety of membrane integrity assays. Evidently, sperm membrane integrity assays such as eosin alone, eosin-nigrosin, hypo-osmotic swelling test, and SYBR14/PI staining are used to assess sperm vitality but are not sensitive enough for the identification of cell apoptosis caused by early membrane damage. Therefore, annexin V/PI, for the detection of phosphatidylserine externalization, are used to identify early membrane injuries during prolonged liquid storage and to assess the preservation efficiency of human tubal fluid (HTF) with different concentrations of human serum albumin (HAS)[107,125]. Moreover, YO-PRO/PI, another assay that can detect early membrane damage, has been widely used in prolonged non-freezing storage of animal sperm [137]. *Pisum sativum lectin* is used for detecting the acrosome integrity, which is often combined with acrosome reaction to assess acrosome function [5].

DNA damage is one of the detrimental effects which causes serious consequences and probably occurs during liquid storage. Similar to the detection of oxidative stress, there are a variety of tests used for the measurement of the genetic status of sperm. These tests include terminal deoxynucleotidyl transferase dUTP nick end labeling (TUNEL), Comet assay, DNA oxidation reflected by 8-OHdG, the sperm chromatin structure assay (SCSA), nuclear protein composition, sperm nuclear maturity test, and sperm chromatin dispersion (SCD) [83]. To date, sperm DNA fragmentation index is the most-frequently used parameter to detect the DNA damage of sperm during prolonged incubation [139]. Sperm DNA fragmentation, measured by SCSA, was shown to increase in a time-dependent manner after 24 h incubation both at room and body temperature [118]. Likewise, prolonged incubation of prepared normozoospermic samples at 37 °C has been found to be associated with higher rates of sperm DNA fragmentation using SCD assay [5,13]. Findings also concluded that the prolonged sperm incubation (5 h) leads to a higher chromatin condensation and to a significantly increased number of DNA strand double breaks but with no influence on fertilization rates [103]. However, a study demonstrated that the level of DNA fragmentation after cryopreservation and storing at wet ice for 24 h were significantly lower than when storing at room temperature for 24 h [126]. Although transportation has no effect on DNA integrity, cooled semen samples stored for 48 h caused a much greater increase in DNA fragmentation index [140]. On the contrary, spermatozoa washed in Modified Ham’s F10 twice and resuspended in IVF fertilization medium can maintain their DNA integrity by using the acridine orange staining method, for up to 12 days at room temperature or refrigeration conditions [107]. Since many studies have highlighted the increase of sperm DNA fragmentation during prolonged storage, it should be an indispensable indicator in assessing the effect of sperm preserved in the liquid state. Moreover, short-term ejaculatory abstinence (1 day) caused a significant increase of DNA-fragmented spermatozoa after 6 h prolonged incubation; thus, such spermatozoa should be used immediately after collection [141]. Although the effect of prolonged non-freezing storage of human sperm on DNA integrity needs more investigation, it is suggested that processing semen samples before storage and then preserving at 2–5 °C helps to stabilize DNA integrity.

Obviously, other sperm function tests function as important indicators of liquid preservation, such as the penetration of zona-free hamster eggs [110]. More studies are needed for exploring capacitation and phosphorylated-related sperm changes during the prolonged non-freezing storage. For the capacitation period, it is found that a long-term capacitation (4 h) results in better sperm quality, inducing a higher percentage of cells with tyrosine phosphorylation and a redistribution of lectin-binding sites [142]. A restricted form of apoptosis was found in extended culture and may increase DNA fragmentation while have negligible effects on the intracellular signaling events related to the capacitation and acrosome reaction [5]. Even though sperm motility, morphology, and other sperm parameters are within the normal range, a comprehensive evaluation should be employed for better verification of extenders to avoid some important omissions. The fertility potential of spermatozoa might decline prior to the decrease of some routine parameters [102]. In addition, the selection of sperm parameters should be based on the purpose of liquid storage, for example, diagnosis or IVF. For the citrate-yolk buffer extender (CYB) developed by R.J Aitken to maintain sperm parameters for delayed diagnosis, aspects of semen quality assessed included viability, motility, hyaluronate penetration, acrosome reaction, and ROS generation. To examine the ability of the electrolyte-free solution (EFM) to preserve the human spermatozoa for IVF, motility, morphology, acrosome status, and especially genetic integrity have been tested one after another. In the recent years, many advanced techniques have been developed, remarkably improving the accuracy, speed, and efficiency in the analysis of many sperm quality parameters. For instance, 3D morphology measurement instead of traditional fixation and staining can capture the changes of sperm structure more precisely [143]. To explore the impact on genetics, compared with DNA fragmentation measurement, telomere, DNA and RNA methylation, and histone modification analysis techniques are able to detect more specific and imperceptible genetic alterations [144,145,146]. The 24 h incubation post-ejaculation affected both sperm DNA methylation and integrity of mouse spermatozoa [146]. NGS provide the most comprehensive and accurate genetic information [147]. The above-mentioned advanced sperm analysis techniques will be helpful to better assess the effect of human sperm liquid storage.

### 4.2. Pregnancy Outcome

Much research that focuses on sperm parameters provided insight on the effect of extended non-freezing storage of human spermatozoa, but the effect of that on clinical fertility outcomes is less studied. Obviously, it is much more difficult to acquire pregnancy outcomes than sperm parameters. However, the time interval between semen collection, processing, and insemination is often clinically different, offering a perspective for investigation. As shown in Table 2, many clinical retrospective cohort studies have been conducted to compare IUI and IVF/ICSI outcomes of so-called delayed insemination with immediate insemination. Home collection of semen samples has been proven to be safe and effective when the time to semen processing is no more than 2 h [8]. Extended preservation of sperm samples at RT or 37 °C also has no effect on clinical pregnancy rate [148]. However, effect of the prolonged incubation time yielded inconsistent results. Jansen et al. reported that time interval within 24 h between semen collection and IUI did not affect pregnancy outcome [149]. In other retrospective studies, delaying IUI to just 1.5 h after semen collection compromises the pregnancy outcome and IUI should be performed as soon as just after processing [150,151]. However, a prospective multicenter cohort study revealed that sperm preparation time in the range of 40–80 min may result in the optimal pregnancy rate [152]. When it comes to ICSI, several studies observed that no adverse effects were detected on the reproductive outcomes within 5 h [9,12,103]. Overall, it suggested that performing IUI or IVF/ICSI immediately after semen collection may be preferable, but the delayed insemination is apparently a feasible alternative for men who are unavailable to provide sperm on the day of oocyte retrieval [37].

As the only FDA-approved commercial reagent for non-freezing storage of human spermatozoa, Refrigeration Medium (TEST-yolk buffer) has been applied in the preincubation of spermatozoa over the last 30 years and proven to favor IUI and IVF outcomes [160]. However, TEST-yolk buffer was mostly used because of its ability to enhance the sperm penetration rate but not to extend lifespan in vitro. Hence, spermatozoa are usually incubated in TEST-yolk buffer for a short period, within 2 h, and subsequently washed for IUI or IVF. In a prospective multicentre randomized trial, the pregnancy rate of IUI was significantly higher in semen preparation by the swim-up with TEST-yolk buffer incubation than in the standard swim-up procedure, especially in patients with unexplained infertility [154]. The same favorable effects of the TEST-yolk buffer were observed in IVF outcomes [155]. Moreover, the TEST-yolk buffer may be superior to other types of culture media such as BWW [158]. Notably, there are a few studies that report the fertilization outcome of sperm maintained in extenders for a longer time, which shows promise for more clinical applications. Paulson et al. demonstrated that compared with a standard swim-up method, the refrigeration of sperm in a TEST-yolk buffer for 24 h following DGC results in a higher in vitro fertilization rate among suspected male factor patients [157]. Furthermore, a similar pregnancy rate was obtained between semen specimens diluted in TEST-yolk buffer immediately used for IUI and that stored for 24 h at 5 °C using a shipping system before being used for IUI. However, this result was based on the premise that the total motile sperm used for IUI was comparable [51]. Remarkably, a favorable pregnancy outcome including delivery date, birth length, and weight was obtained using human spermatozoa preserved in another semen extender, EFM, at 4 °C for up to 2 weeks [10]. The above-mentioned results suggest that the fertility potential of human spermatozoa is able to be maintained at non-freezing conditions in vitro safely and effectively for at least several days.

## 5. Storage Time and Temperature

**Figure 3:**
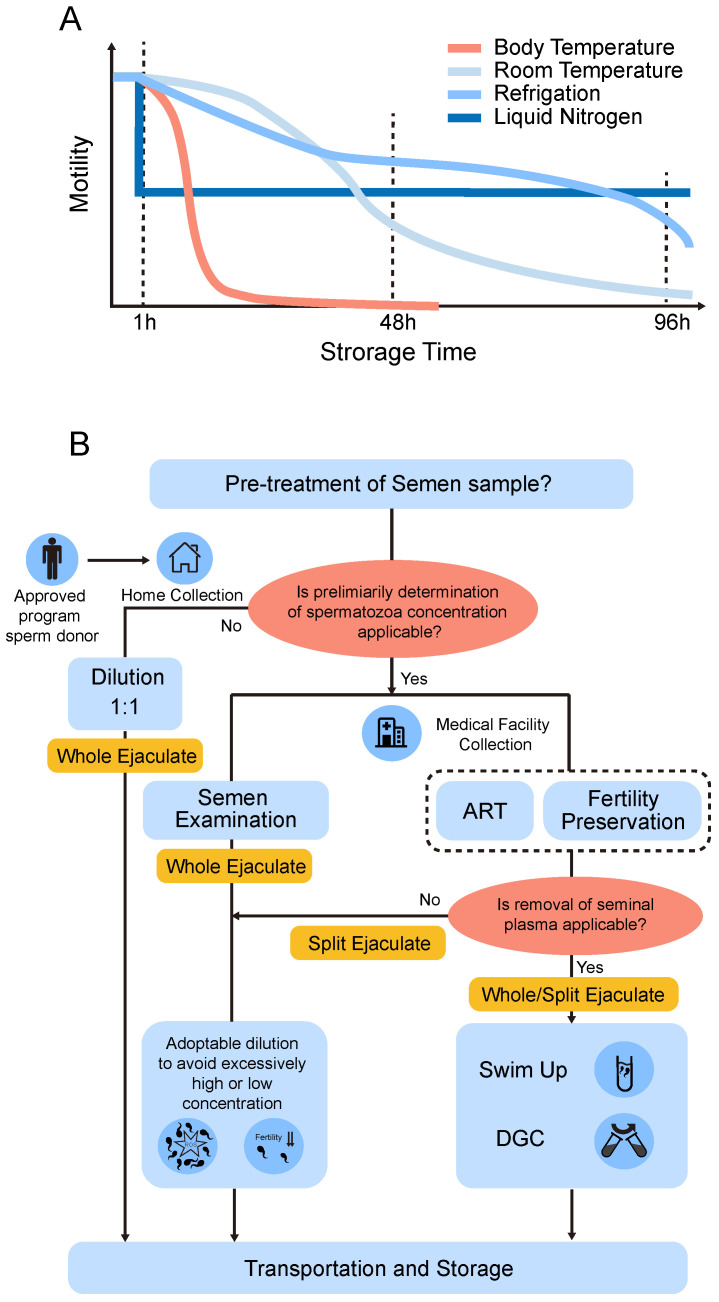
Preserving conditions and strategies of liquid storage of human sperm. (**A**) A general decreasing trend of sperm motility over time when stored at body temperature, room temperature, refrigeration conditions, or liquid nitrogen in vitro. (**B**) Schematic overview of a possible workflow when making decisions about whether the pretreatment of semen samples before liquid preservation should be conducted or not.

Storage duration and temperature are two of the most fundamental concerns when preserving human spermatozoa in the liquid state. In spite of the varying experimental designs and inconclusive study results, a preliminary conclusion can be drawn in this review (Table 1, Figure 3A). In any case, human sperm preserved in the liquid state should not exceed 96 h of preservation time. Semen samples should not be kept at 37 °C after liquification. When storing sperm within 48 h, the storage temperature is preferred to be 22–25 °C, while when storing sperm for more than 48 h, 2–5 °C is preferable.

In general, the limit of the liquid preservation time is approximately one month according to the reports by Jonathan M. Riel et al., and that viable sperm and motile sperm can be detected after preservation in cold electrolyte-free medium (EFM) for up to 4 weeks and 7 weeks, respectively [11,34]. However, a >50% motility recovery of sperm diluted in TEST-yolk buffer was obtained in refrigeration conditions for no more than 1 week [21]. When it comes to the storage temperature, it should be defined as the temperature that human spermatozoa are stored after liquification or pre-treatment. Makler et al. stated that sperm survived up to 24–48 h when stored at 23 °C, while at body temperature, their survival in vitro was much shorter and rarely extended beyond 12 h without any treatment in an early study [4]. It has also been proven by much research that spermatozoa stored at hypothermia (0–35 °C) are superior to those stored at normothermia when the incubation time exceeds 1 h [88,161]. The longer the duration needed, the lower the adoptable temperature. Hypothermia restricts the metabolic rate of the spermatozoa during storage for the purpose of reducing detrimental effects such as the depletion of ATP and the production of ROS. However, lower temperatures and the chilling process probably induce cold shock in spermatozoa, an abrupt lipid membrane phase separation which permanently impairs the fertility potential of sperm [80]. Therefore, hypothermic preservation comes with risks and must be chosen carefully in order to achieve the optimum storage efficiency [162]. Therefore, three different storage temperature ranges, ambient temperatures (25 °C), 10–17 °C, and refrigeration temperature (2–5 °C) are employed in most research, which is associated with the susceptibility to the cold shock among different species. For instance, boar sperm must be stored at 17 °C due to its extreme vulnerability to low temperatures [163,164,165]. The ram or stallion sperm are allowed to be stored at lower temperatures (2–5 °C), while the decline of sperm quality is inevitable [16,166]. For prolonged liquid storage of human sperm, ambient temperature and refrigerated temperature are chosen most. When storing sperm within 48 h, the storage temperature is preferred to be 22–25 °C while when storing sperm for more than 48 h, 2–5 °C is preferable (Figure 3A) [107]. Yet we have not found studies that explored the human semen storage at 16–17 °C, considering the high resistance of human sperm to low temperatures, we speculated that human sperm could be effectively preserved at 16–17 °C. Liquid storage instead of cryopreservation is strongly suggested when spermatozoa are ready to use within 48 h. It was first reported by Jacques Cohen, who compared the quality of human semen kept at 5 °C with that of freshly ejaculated semen and semen cryopreserved in liquid nitrogen [32]. Compared with cryopreservation, refrigeration not exceeding 48 h with the aid of TEST-Yolk buffer obtained results in comparable and significantly better motility and hypoosmotic viability test in a normospermic and infertile samples, respectively [106,167]. Considering the high resistance of human sperm to cold shock, liquid storage of human semen samples at 2–5 °C should be given more attention. The benefits of lowering the temperature to 2–5 °C is more than maintaining the sperm viability and motility, which is much easier to control during the shipment of sperm samples; even a simple container including an ice bag could meet the requirement. However, it is reported that cooling rate plays an extremely important role in the efficiency of preservation at refrigeration conditions. For the preservation of human spermatozoa, the cooling rate should be slowed down at least −0.5 °C/min. For the livestock spermatozoa such as stallion spermatozoa, the cooling rate should be controlled below even −0.05 °C/min. Otherwise, an irreversible cooling injury may occur. The realization of such a slow chilling process requires specially made equipment such as the freeze container, which may not be readily available. Moreover, it is worth noting that with the storage time increasing, the fertility dropped inevitably whether the sperm are stored at 4 °C, 15 °C or ambient temperature (25 °C), whereas some of the sperm parameters such as motility can be maintained during the whole period of storage [102]. Therefore, the choice of duration and temperature during storage is much more complex in real clinical scenarios compared with that in the laboratory. Determination of storage time and temperature highly depends on the objective of the liquid storage. For objectives that demand reflecting on the true state of fresh semen such as the clinical diagnostic test, ambient temperature should be chosen with a higher priority, resulting in shorter preservation or transportation times. In terms of delayed ART or cryopreservation, cold storage is more appropriate. A slight loss of sperm quality is worthwhile as long as it can benefit more patients who require fertility preservation from remote sites. However, studies have shown a significant increase of DNA fragmentation over time post ejaculation in different temperatures, further reminding the potential risks of prolonged liquid storage [126].

Optimization of storage duration and temperature are also helpful for selecting ejaculated and testicular sperm for successful ART outcomes. It is reported that the sperm cells after devitrification showed better viability and mitochondrial membrane potential when incubated at RT than at 37 °C [15]. For testicular sperm retrieval, in vitro culture for 24 h is beneficial to pregnancy outcomes [168]. Findings state that the morphology and motility of fresh or frozen-thawed testicular spermatozoa from the obstructive azoospermic patients were significantly improved after being incubated for 72 h before IVF manipulation whereas is uncertain in non-obstructive azoospermia [169]. Further study demonstrated that the ideal cultural time and temperature for testicular spermatozoa was 24 h and 25 °C [14]. The result was practical for liquid storage of azoospermic TESE samples.

## 6. Pre-Treatment of Semen

### 6.1. Semen Processing and the Effect of Seminal Plasma

Apparently, except for the storage time and temperature, semen preparation before liquid storage is another issue of great concern. Semen processing technologies such as swim-up and density gradient centrifugation (DGC) have favorable effects on the extension of sperm longevity [34,121,170,171]. Although some studies found DGC itself including multiple steps, washing and resuspension of human spermatozoa during these periods may increase the level of oxidative stress and impair sperm motility, their merits far outweigh demerits: the removal of dead sperm, leukocytes, and seminal plasma after DGC got rid of the main source of ROS, resulting in a significantly higher percentage of progressively motile sperm than initial semen [172,173]. There is no doubt that the addition of extenders benefits sperm preserved in the liquid state. Nevertheless, the use of extenders entirely in place of seminal plasma during the liquid storage remains controversial [174,175,176,177]. Despite seminal plasma being essential for sperm, such as for preventing immediately capacitation, studies suggest that sperm resuspended in extenders after removing seminal plasma may improve the performance of liquid storage [11]. However, it is unclear whether the removal of seminal plasma is responsible for this improvement. Leukocytes and immature and abnormal sperm cells seem to have more detrimental effects on liquid storage than seminal plasma [178]. Findings suggest that seminal plasma must be removed after liquification in ART to mitigate DNA damage [127]. Interestingly, there is no significant difference between spermatozoa stored in the normal seminal plasma and that stored in extenders after DGC, which suggests that seminal plasma can be divided into “good” and “bad”; the latter is more common in oligoasthenzoospermia patients [119,179]. Spermatozoa left in the “good” seminal plasma maintain the sperm quality for a longer time, but that in the “bad” seminal plasma deteriorate quickly. The “bad” seminal plasma may have an abnormal pH and contain more harmful substances such as higher level of insulin-like growth factors (IGF) and prostate-specific antigen (PSA), as the consequence of accessory sexual gland diseases such as chronic prostatitis [180]. Some seminal plasma proteins were also found to be associated with sperm swimming velocity and hyperactivation [181]. In terms of the role of seminal plasma and the split ejaculate method, one of treatments to avoid the negative effect of it with the potential for the non-freezing extended preservation of human sperm is discussed in depth in the next section.

### 6.2. The Split Ejaculate Collection for Non-Freezing Storage of Human Sperm

Seminal plasma consists of secretions mainly from the testis and epididymis (~10%), prostate (~25%), and seminal vesicles (~65% of semen volume). It provides nutrition for spermatozoa and plays important roles in the regulation of semen coagulation and liquefaction, sperm motility, and fertilization [182]. Seminal plasma carries substances that stimulate sperm motility. On the other hand, exposure to seminal plasma for a longer time may be detrimental to sperm parameters, which is attributed to the existence of seminal vesicular fluid [183]. It is well established that the seminal vesicle secretions impair sperm functions and survival by increasing osmolarity and damage the zinc-dependent chromatin stability of sperm. Thus, a better sperm survival and fertility success in ART would be achieved by avoiding sperm contact with the hostile seminal vesicular fluid [184]. Actually, seminal plasma is a heterogeneous mixture of secretions that do not exist within the body before being expelled. Due to the anatomical structural differences, the first one-third part of the ejaculate contains the majority of spermatozoa suspended in the prostatic fluid, and the second two-third part of the ejaculate contains the seminal vesicular fluid [185]. It is proved that the first ejaculate fraction is superior to the rest fractions in terms of total sperm count, percentage of active motility, and chromatin stability, which can be obtained by an easy split ejaculate collection [186,187,188]. In natural sexual intercourse, the initial fraction of the ejaculate that is dominated by sperm-rich prostatic fluid is likely to come into contact with cervical mucus without any significant contact with the rest of the ejaculate that is dominated by seminal vesicular fluid. In contrast, in the laboratory setting until now, the entire ejaculate is collected in one container for both semen examination and ART. Hence, the call for the use of split ejaculates for ART, which contains the majority of spermatozoa with high motility and is closer to the natural conception, has been around for a very long time, whereas the fertility potential and pregnancy outcomes of the split ejaculate method need to be further assessed [184,189,190].

Considering the ejaculate used for immediate ART discards the seminal plasma after collection, the use of split ejaculate in prolonged preservation of human sperm for delayed ART should be highlighted, which so far has not been reported. Although there is no worry regarding the decline of spermatozoa left in the acidic prostatic fluid for an extended period, findings state that spermatozoa ejaculated in the two halves of the ejaculate show a better viability and motility in the first than in the last half for at least 4 h, suggesting the damage is limited [191]. It is reasonable to speculate that the addition of semen extender into the first fraction of ejaculate would further promote the preservation effect. However, the whole ejaculate should be collected for laboratorial tests in order to obtain a complete evaluation of the sperm and accessory sex glands.

In summary, it is preferable to perform semen processing before prolonged non-freezing storage in vitro for delayed ART or fertility cryopreservation, utilizing the extender instead of the seminal plasma. However, spermatozoa without seminal plasma are not applicable for clinical laboratory testing and the pre-treatment of semen cannot be completed at home. Semen samples should be diluted in semen extenders if the reproductive medical facility is not readily available. In addition, collection of the first fraction of the split ejaculate following extender dilution may further benefit the outcome of prolonged preservation for ART but it is not suitable for semen examination. When it comes to sperm donation, it should be noted that men approved to be program donors can collect semen samples at home and then ship them to the sperm bank using the “donation kit” directly with no other pre-treatment. The pre-treatment of semen depends on more than the clinical purpose and collection sites for prolonged liquid storage (Figure 3B).

## 7. Semen Extenders Used in Non-Freezing Storage of Spermatozoa

**Figure 4:**
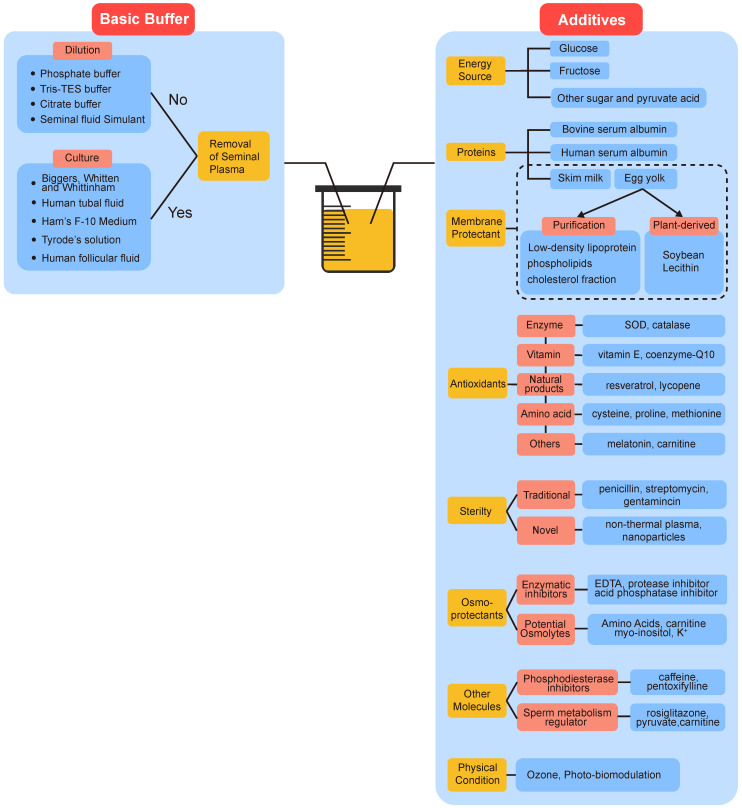
The components of extenders used in liquid storage of spermatozoa. They are divided into basic buffers providing stable physiological environments, and various additives help to mitigate oxidative stress, protect the membrane, resist contamination etc. Please see the main text for further details and interpretations.

The addition of substances such as nutrients and protectants to maintain spermatozoa during the extended storage is essential, known as the extender (or culture medium). Nowadays, most extenders utilized for liquid storage of sperm are typically composed of two parts: buffers, providing a suitable physical and chemical environment for sperm, such as stable buffer capacity and osmolality pressure, similar to natural cases; and additives, such as antioxidants and membrane stabilizers, offering support to extracellular and adverse intracellular factors (Figure 4). Compared with cryopreservation, sperm stored in the liquid state are confronted with more complicated risk factors. Chief among them are negative effects of sperm metabolism, cold shock, and bacterial contamination.

### 7.1. Basic Buffer and Dilution Ratio

The appropriate pH and osmolarity of semen can be maintained via adding physiological buffers during liquid storage, which comprise the basic substance of extenders. The roles of physiological buffers are more important in stabilizing pH than in osmolarity, which may not be enough to fully counteract the increase of osmolarity with prolonged time. The osmotic shock of sperm can be further protected by the addition of osmoprotectants in the extender (this is discussed in the next section). As mentioned above, the phosphate buffer with egg-yolk was the extender initially used for longer preservation of bull spermatozoa [18,19,20]. This extender forms the fundamental formulation of follow-up modification and substitution. Continuing work developed sodium citrate, carbonate, Tris-buffered materials, and nonionic materials as basic buffers for the preservation of animal spermatozoa [192,193,194]. However, these substances were not superior in the long-term storage of spermatozoa in the liquid state. In the 1970s, along with several zwitter ion buffers between pk 6 and 8, which were available to the biologist, Tris with N-Tris (hydroxymethyl) methy-2-amino ethane sulfonic acid (TES) was found to be the most satisfactory buffering system for the preservation of bull spermatozoa [195]. Since then, the Tris-TES buffer became the most widely used basic buffer system in sperm cryopreservation and liquid storage [21]. Tris-TES’s replacement of other buffers together with egg-yolk was taken for granted, called TEST-yolk, showing many benefits to the viability, fertilizing capacity, and storage potential of human spermatozoa and it has also been used in sperm function testing and sperm preparation for clinical IVF [196,197]. It was summarized that the main beneficial effects of TEST-yolk are attributed to the yolk component, and the Tris-TES component probably acted synergistically [198]. Few harmful consequences of the TEST-yolk have been reported, whereas the molecular mechanism of TEST-yolk exerting its effects remains unknown [160]. Except for TEST, citrate was another chemical composition most frequently used in sperm storage. It is rich in human semen and a considerable contributor to the buffer capacity of the semen [56,192]. Citrate buffer in place of phosphate buffer showed better motility in long-term liquid storage of bovine spermatozoa [192]. However, there have been many substitutes for TEST-based or TEST-citrate-based extenders used in cryopreservation, which show better post-thawing efficiency. To this day, they are irreplaceable and predominate reagents employed in the liquid storage of semen samples, and we discuss them in detail in the following sections (see later text).

Except for TEST-based or TEST-citrate-based extenders, there are many buffers which are reported to be employed for in vitro storage of human spermatozoa in the liquid state, where Biggers, Whitten, and Whittingham (BWW) media, Human tubal fluid (HTF), Ham’s F-10, and Tyrode’s solution are frequently used. The components of them are similar, mainly including sodium, potassium, magnesium salts, and monohydrate as well as dihydrate phosphate salts. All of them are bicarbonate buffers as a simulant of the real acid-base homeostasis system in living tissues. For example, the composition of HTF medium mimics the physiological environment in which the early human embryo is developed [199,200,201]. On the day of use, stock solutions of the above-mentioned buffers are usually supplemented with glucose, bovine serum albumin (BSA), or antibiotics to prepare the fresh complete culture medium. These buffers are initially developed for gametes’ and embryos’ in vitro handing and cultured so that they have been commonly used in the examination and processing of human semen for many decades [202]. For instance, BWW media are used in the zona-free hamster oocyte penetration test and assessment of acrosome reaction. However, in studies which pertain to the liquid storage of human spermatozoa, they are usually act as a substitute for seminal fluid in the control group, which suggested the safety but not the superiority of these media in maintaining the human spermatozoa in vitro, as mentioned previously. BWW is rarely reported to be involved in preserving human sperm. HTF medium was used for delayed ART or cryopreservation of human sperm but failed in the competition with the TEST-Yolk buffer and electrolyte-free medium [34,45]. Compared with HTF medium, Ham’s F10 medium is more frequently used as the in vitro washing and culture medium to explore the effects of liquid storage on ejaculate and testicular sperm parameters at different temperature and time interval [14,107]. Notably, many studies found that human spermatozoa cultured in Ham’s F10 medium enhanced the risk of peroxidative and DNA damage while this detrimental effect seems to be mitigated by adding antioxidants such as vitamin E [5,116,203,204]. Ham’s F10 medium is not considered as the optimal culture medium of human spermatozoa and the use of it is not recommended in the newest version of the WHO’s guidelines [38]. A modified high-potassium Tyrode’s medium is another buffer that is used for the liquid storage of mammalian spermatozoa, including human [205]. It is often mixed with Kenney’s extender (KE), which is a commonly used semen preservative containing skim milk, glucose, and antibiotics [206]. The sperm of mammalian animals diluted in KM or KMT could be effectively preserved for 48 h in refrigeration condition after centrifugation [174,207]. Additionally, a real human-derived medium, human follicular fluid (HFF) from female oocyte, showed substantially beneficial effects on prolonged incubation of human spermatozoa [111]. It significantly improved the motility of sperm and reduced DNA fragmentation index [128]. Nevertheless, addition of HFF in extenders for mass production is difficult. Overall, these solutions exhibit an excellent buffer capacity and are appropriate for cell or embryo culture, but their effects are suboptimal in terms of the liquid storage of human spermatozoa compared with TEST-based or TEST-citrate-based buffers. In addition, these cultural mediums may demonstrate suitability for the incubation of spermatozoa in vitro; however, their use usually requires discarding seminal plasma, more useful for the short-term preservation of testicular spermatozoa.

If seminal plasma is not completely removed during the prolonged non-freezing storage period, the semen extender also serves as a diluent. Dilution changes the sperm concentration, does not change the percentage of motility, and may increase their velocity [208]. Studies have shown that sperm concentration significantly affects many sperm parameters in liquid storage and cryopreservation [209,210]. Therefore, optimizing the dilution ratio of the initial semen samples to extenders possibly improves the preservation efficiency. To date, the dilution ratio of 1:1 (*v*/*v*) is chosen in most studies and commercial products, while the reason for this choice has not been exactly demonstrated. Sperm concentration varies in individuals drastically, causing a tremendous difference in the rate of metabolism and accumulation of ROS, impacting their post-ejaculated lifetime. Higher sperm concentrations were found to increase oxidative stress in liquid bull semen [211]. In contrast, lower sperm numbers after 48 h of liquid storage reduced the fertility of bull semen, which is inferior to the frozen-thawed semen [212]. Moreover, no substantial benefit to sperm quality was seen from the reduction of sperm concentration during liquid storage of goat spermatozoa [213]. On the basis of these studies, the maintenance of human sperm concentration is supposed to be a preferable option. An extensive dilution should be avoided, and it is sensible to develop extenders demanding less medium per application to minimize the dilution of semen samples in the case of low concentration [214]. Determination of the sperm concentration is helpful to improve liquid storage efficiency when the original sperm concentration is able to be examined. However, when unable to measure the sperm concentration, a 1:1 sample: extender ratio should be conducted.

### 7.2. Additives

#### 7.2.1. Energy Sources, Proteins, and Antibiotics

Given that storing semen in hypothermia is strongly recommended, spermatozoa metabolic rate and oxygen demand are substantially reduced under hypothermic conditions. However, they are not totally arrested, and sperm cells might be subjected to rapid ATP depletion. Therefore, the addition of energy substances is essential for prolonged liquid storage of semen. ATPs used by spermatozoa derive mainly from the metabolism of glucose and fructose in seminal plasma. Other sugars and pyruvate acid also play a role in ATP production. Fructose is secreted by the seminal vesicle and its concentration varies among individuals. The concentration of fructose in fresh human semen is approximately 2–3 mg/mL and is drastically reduced to nearly 1/10 of its initial concentration after incubation at 37 °C for 3 h. Fructose is the predominant sugar in seminal plasma, which is 50-fold higher in concentrations than glucose. Sperm prefer to consume glucose, which suggests that glucose is actually the major source of glycolytic energy in human sperm. This may be attributed to the high-affinity constants of sperm hexokinase for glucose. Sperm motility is found to be dependent on glucose concentration and maximum motility occurs in glucose concentration equal to that in cervical mucus at midcycle. In addition, glucose showed superiority in supporting acrosome reaction and hyperactivation than other sugars. Thus, glucose is more frequently used in extenders prepared for human or animal sperm. Nevertheless, these two molecules can be used as the substitute for another one. Addition of glucose in extenders combined with hypothermia might exert a synergetic effect on long-term preservation in the liquid state [120,215].

Protein is an essential constituent for the in vitro storage of sperm, which exerts many protective effects such as stabilizing osmolarity, resisting oxidation, and maintaining membrane integrity [216,217,218]. Proteins in semen are broken down into amino acids continuously in the hours following ejaculation, so it is required to be supplemented in extenders. Albumin consists of approximately 1/3 of total proteins in semen, so human or bovine serum albumin are most frequently used in IVF culture media and semen extenders. However, when egg yolk or skim milk presents, there is no need to add additional human or bovine serum albumin in extenders. To our best knowledge, formulations with buffers plus human or bovine serum albumin are not superior to TEST-yolk based extenders.

Antibiotics have a long history of application in extenders to prevent semen from microorganism contamination, which leads to the deterioration of spermatozoa when a longer preservation time is needed. Penicillin and streptomycin have been shown to effectively control bacteria and improve fertility in bulls in the 1950s [219]. Adding antibiotics into extenders is effective; nevertheless, sterile techniques are the basis of inhibiting the growth of bacteria. Notably, it is very hard to achieve the aseptic condition during the practical collection and transportation of semen samples, especially in patients with no experience of sterile operation. Therefore, the addition of antibiotics in extenders is highly recommended. A recent study showed that non-thermal plasma has the potential to be a novel replacement to antibiotics for semen preservation because of its significant antimicrobial effects [220]. Although an appropriate exposure to non-thermal plasma was observed to have no negative effects on the human sperm structure and function, its clinical use should be further investigated carefully.

#### 7.2.2. Membrane Protectant

Egg yolk was the first substance shown to exert a membrane-protective function used to prolong the liquid storage of spermatozoa. However, the inspiration for choosing the egg yolk is very simple, “the naturally-occurring materials should be first tested and the egg is the counterpart of spermatozoa”, reported by Phillips [19]. The improvement of egg yolk has been well-documented in animals, and the similar effects in the preservation of human sperm were proven in the 1980s [21,221]. The mechanism of the beneficial effects of egg yolk has been subjected to intensive research. Currently, egg yolk is believed to predominantly act as a protectant of sperm cell membranes during cooling and freezing. The low-density fraction contained in egg yolk was firstly discovered as a component to protect sperm during freezing [222]. The abundant lipoprotein and phospholipid fraction of egg yolk play an important role against cold shock via minimizing lipid loss from the sperm during cooling. Different techniques and varying lipid constituents of egg yolk have demonstrated the binding of the lipid to the spermatozoon membrane, generally accepted as being due to active constituents in the low-density lipoprotein fraction [223,224,225]. Phospholipids, particularly those in egg yolk, protect sperm to some extent from cold shock and also prevent increased calcium flux into the sperm [92]. However, some of the substances which exist in yolk granules, such as high-density lipoproteins (HDL) and minerals, may inhibit sperm respiration and lead to a decrease in sperm motility. Moreover, the sanitary concerns drive scientists to search for egg yolk alternatives. Apparently, chemical-defined extenders are preferred [99,226]. There are mainly two strategies to develop better membrane protectants instead of the whole egg yolk: one is the use of a more purified or certainly chemical-defined substance contained in egg yolk; egg yolk plasma and saline-soluble fraction were shown to have the potential to replace the whole egg yolk in livestock freezing extenders [227,228]. Since the protective action of egg yolk is largely attributed to the low-density lipoprotein, phospholipids, and cholesterol fraction existing in it, the effort towards its application in preservation extenders has never ceased. Low-density lipoprotein could easily be extracted from the whole egg yolk and exhibit a marked improvement in motility parameters [224,225]. Incubation with cholesterol-loaded cyclodextrins prior to cryopreservation of semen was shown to lead to higher cryosurvival rates and post-thawing sperm quality, whereas fertility rates were not improved, perhaps because of the longer capacitation time [229,230]. The other is the usage of plant-based extenders in substitution for egg-yolk extenders, and these have become a trend in sperm preservation for decades [231]. The same as egg yolk, soybean lecithin was first used in livestock sperm cryopreservation and has undergone rapid development [226,232,233,234]. Subsequently, the effect of the soybean lecithin-based extender was evaluated in liquid storage of buffalo, ram, and ovine semen in comparison with milk and egg-yolk-based extenders [98,100,101,235]. The optimal content of soybean lecithin in storage medium was between 0.5% and 2% (*w*/*v*) [236]. Soybean lecithin in the place of egg yolk as a freezing extender for the cryopreservation of human sperm also has been tried [237]. Compared with egg yolk, no significant differences were found in several important sperm parameters after cryopreservation with soybean lecithin [238]. However, it seems that the advantage of soybean lecithin application achieves no more than the elimination of biosecurity concerns, and few studies report the improvement of preservation. This may explain why the interest in liquid storage of human sperm using soybean lecithin-supplement medium is rare. Nevertheless, the potential of soybean lecithin in the liquid storage of human sperm should not be ignored. Recently, phosphatidylcholine extracted from soybean lecithin was shown to have potential as a substitute for egg yolk in human sperm cryopreservation [239]. Along with the application of nanotechnology in semen preservation, it freezing medium containing liposome-encapsulated soy lecithin and cholesterol has been reported to have a better cryopreservation efficiency of human sperm than conventional egg yolk [240]. The advantage of liposome and soybean lecithin nanoparticles are also shown in frozen animal semen [233,234]. Obviously, these innovations of membrane protectants in semen cryopreservation could pave the way for the improvement of liquid storage technology.

#### 7.2.3. Antioxidants

Numerous studies have emphasized the great importance of oxidative damage during the liquid storage of spermatozoa. In addition to hypothermia, preservation capable of inhibiting ROS production, the supplementation of antioxidants in extenders is considered as playing a major role in mitigating oxidative stress. Many antioxidants have been used in the liquid storage of sperm and antioxidants applied for liquid storage or cryopreservation are highly similar. These antioxidants, including their classification, mechanism of action, and efficiency, have been subjected to intensive research and well summarized in previous reviews. It is found that some of the antioxidants are highlighted as storing sperm in the liquid state. Vitamin E is regarded as being especially suitable for sperm preservation due to its superior capacity for protecting the sperm plasma membrane from lipid peroxidase. Therefore, the addition of vitamin E is strongly recommended in CYB medium developed by RJ Aitken [22]. However, adding vitamin E in Ham’s F10 medium cannot improve the maintenance of human sperm vitality and motility during the liquid storage, which is reflected in results of livestock [84,116]. L-carnitine is a small peptide enriched in epididymis, acting as both a key molecule in β-oxidation of fatty acids and an effective antioxidant. The addition of L-carnitine in extenders showed a favorable effect on many mammalian species either at room temperature or refrigeration condition, which suggested that it has potential to be a promising antioxidant in the liquid storage of human sperm [241,242]. Coenzyme-Q10, also playing a key role in ATP synthesis and energy production, is observed to be highly positively correlated with sperm parameters [243]. Spermatozoa of asthenospermic patients cultured in Ham’s F10 medium with Coenzyme-Q10 incubated for 24 h showed a significant increase in motility [244]. Notably, a common issue faced by these lipid-soluble antioxidants is that the use of cosolvents such as DMSO or absolute ethanol are required in spite of low concentrations (<1%), which are toxic to spermatozoa. This issue can be eliminated via using water-soluble antioxidants. For example, Trolox is a water-soluble vitamin E analogue which exhibits comparable preservation efficacy. Mitoquinone (also named as MitoQ) is a derivative of Coenzyme-Q10, which is a water-soluble, mitochondria-targeted antioxidant. It is concentrated in mitochondria, 100–1000 times higher than the concentrations of Coenzyme-Q10 and provides a strong antioxidant capacity. Findings have demonstrated that the addition of Mitoquinone in freezing extenders significantly improved the post-thawing human sperm quality [245]. Melatonin, a hormone, has also emerged as a potent water-soluble advanced antioxidant. It has been employed in extenders for the liquid storage of human spermatozoa [246]. In addition, melatonin has been revealed to be able to alleviate the heat stress-induced oxidative stress and apoptosis in human spermatozoa after incubation for 24 h at 37 °C [135]. Since egg yolk already provides abundant antioxidant substances including vitamin E, TEST-yolk-based extenders might not need to provide supplementary antioxidants. In addition, the use of antioxidants in combination with another extender, whether exerting synergetic effects or not, requires further exploration. Recent studies reveal that the development of nanotechnology and drug delivery technology, such as vitamin E nanoemuisions or liposome encapsulation, have the potential to further optimize the efficiency of antioxidants in the liquid storage of human sperm [240,247,248].

#### 7.2.4. Osmoprotectants

One important factor that contributes to the decline of sperm quality since the time of ejaculation is the dramatic increase of semen osmolarity. To solve this problem, the dilution of semen with isotonic extenders aids to correct the abnormal osmolarity. Besides, some substances called osmoprotectants have been used to further protect sperm from osmotic injury. According to the mechanism of action, these osmoprotectants can be divided into mainly two categories. It is believed that the increase of semen osmolarity is mainly attributed to the continued enzymatic activity in seminal plasma. These enzymes such as protease and acid phosphatase cleave large proteins presenting in seminal plasma into choline, amino acids, and other smaller osmotically active peptide fragments, causing hyperosmolarity [22]. Hence, the first category of osmoprotectants comprises some enzymatic inhibitors, for example, EDTA, acid phosphatases, and protease inhibitor. Compared with dilution, these molecules seem to be more effective in hindering the rising of semen osmolarity during the prolonged storage of semen. Findings state that the presence of enzymatic inhibitors assuages the increment in semen osmolarity by 75% after 24 h of incubation [65]. However, they may enhance the risk of oxidative stress and cannot provide further improvements on the presence of egg yolk [22]. In addition, storing semen in lower temperatures may help to maintain osmolarity via decreasing the enzymes’ activity [6]. The second mechanism of action is to enhance the capacity of cell volume regulation of spermatozoa in non-physiological osmolarity by adding potential osmolytes in extenders. As mentioned previously, when extracellular or intracellular osmotic imbalance occurs in cells, including spermatozoa, they have the capacity to maintain the stability of cell volume via efflux or influx of osmolytes. It is observed that a change of osmolarity highly influences the development of embryos, so the maintenance of osmolarity in IVF cultural medium is subjected to intensive research. The addition of organic osmolytes such as amino acids including glycine or L-proline into culture medium has a favorable effect on the in vitro culture of gametes or embryos. TEST-citrate-yolk medium with glycine has been proven to be superior to TEST-citrate-yolk medium. Meanwhile, glycine is supposed to serve as a chelator of toxic heavy metals in semen. The protective role of L-proline is probably due to both its antioxidant and osmoprotectant properties [249]. Many studies report that taurine is a key organic osmolyte in most cells, including human oocytes and embryos, whereas taurine does not appear to play a role in human sperm volume regulation [250]. Besides, organic molecules such as glutamate, carnitine, and myo-inositol, as well as inorganic molecules such as K^+^ and Cl^+^ are important contributors toward semen osmolarity and they have been proven to be effective osmolytes in murine spermatozoa. However, these putative osmolytes showed no effect on human spermatozoa except for glutamate and K^+^. Similarly, some osmolytes such as carnitine and myo-inositol also serve as effective antioxidants in sperm storage. Therefore, glutamate and K^+^ might be the major osmolytes utilized by human sperm [250]. The possible potassium and chloride channels involved in volume regulation of human spermatozoa have been identified in physiological regulation volume decreases [251,252]. However, the effectiveness of glutamate and K^+^ in continuously increasing the seminal plasma osmolarity has not yet been well explored.

#### 7.2.5. Other Molecules

Inhibitors of phosphodiesterase, such as caffeine, pentoxifylline, and hypoxanthine, have been reported to act as beneficial additives in extenders [253]. These motility stimulants function mainly via increasing the concentration of intracellular cAMP, promoting sperm energy metabolism, thus strongly improving motility parameters of spermatozoa with no effect on sperm viability [254]. Pentoxifylline, which is widely used in assisted male reproduction techniques, is well known for its ability to activate immotile testicular sperm before ICSI in azoospermic patients [255]. Caffeine and pentoxifylline supplementation in cryoprotectants is also helpful for human sperm cryopreservation [256,257,258]. The addition of caffeine in extenders has a positive effect on ram sperm preserved at 4 °C for 48 h [259]. However, the effect on the above-mentioned motility stimulants seems to be not enough for maintaining the whole duration of liquid storage [260,261].

In addition to phosphodiesterase inhibitors, there is another type of molecule regulating sperm metabolism that has been proven to be effective in the prolonged storage of spermatozoa. It is observed that glycolysis produces significantly less ROS than oxidative phosphorylation, while the latter is the more efficient pathway employed by spermatozoa [80]. Hence, diverting the mode of ATP production in sperm from mitochondrial metabolism into glycolysis may have a protective effect due to the reduction of oxidative stress. It was found that rosiglitazone, an antidiabetic compound that has this ability, significantly alleviated the time-dependent deterioration of stallion spermatozoa [138]. Two major modulators of oxidative phosphorylation, pyruvate and carnitine, can improve the efficiency of longevity of stallion sperm in the liquid state [242]. Furthermore, in vitro treatment by growth factors such as fibroblast growth factor 21 (FGF21) significantly increased human sperm motility and ATP levels [262]. As discussed in the previous section, although human sperm cells have a different metabolic pattern compared with livestock, it is worth attempting to explore them in storing human spermatozoa at ambient temperatures.

In a recent study, low doses of ozone were found to induce a positive response in the equine sperm kinetics patterns and sperm structure during cooled storage [263]. In addition, preconditioning human semen samples with photo biomodulation before cryopreservation provides a real and substantial advantage, increasing the number of viable and motile spermatozoa [264]. The beneficial effects of both ozone and photo biomodulation treatment may be attributed to the stimulation of a mild oxidative stress in spermatozoa, which upregulates the antioxidant defenses systems in cells [265].

## 8. Typical Extenders Used to Store Human Spermatozoa in Liquid State

To date, there are four extenders which have been developed with the definitive aim of liquid storage of human spermatozoa. Two of them were designed initially for ART, one was for clinical laboratorial testing, and the last one was initially for in vitro research in reproductive health and then exhibited potential for multiple applications. The main characteristics of the above-mentioned extenders are summarized in Table 3.

### 8.1. Refrigeration Medium-TEST-Yolk Buffer with Gentamicin (Commercial Agents)

Since the dilution of human semen samples with TEST-Yolk buffer has proven to be effective in prolonged liquid storage of human spermatozoa, a so-called “Refrigeration Medium” with a few modifications on the basis of the TEST-Yolk buffer was developed by FUJIFILM Irvine Scientific, Inc. USA (https://www.irvinesci.com, accessed on 10 September 2022). To our best knowledge, it is currently the only product for storing semen in a liquid state commercially available in the world, which is CE marked and FDA-cleared. The components of the Refrigeration Medium include TES/Tris to provide buffer capacity, dextrose to provide energy, gentamicin sulfate to resist bacteria, and heat-inactivated egg yolk from specific pathogen-free (SPF) laying flocks to provide multiple protective effects. According to the product inserts, the medium should be added dropwise into the liquefied semen samples until to achieve a 1:1 volume ratio with thorough mixing. The mixture is cooled from 37 °C or ambient temperature to 2–5 °C very slowly and ultimately allows the preservation of sperm fertility potential for up to 4 days. The study brochures showed that sperm motility of samples stored at ambient temperature or refrigerated at 4 °C with the Refrigeration Medium after 24 h and 48 h following ejaculation was better than and comparable to the motility of cryopreserved semen, respectively. However, the Refrigeration Medium did not present significant advantages in motility retention within 48 h in comparison with semen stored at ambient temperature in seminal fluid alone [167]. The Refrigeration Medium may present better performance in 48–96 h, which has been demonstrated by David G. Jaskey et al. [21]. This Refrigeration Medium was designed initially for delayed ART in men with normal semen parameters. Afterwards, the medium was introduced in male fertility preservation due to infertility or cancer, which shows similar cryosurvival rates between samples cryopreserved after shipment of 18 h or samples cryopreserved immediately [24,45]. In summary, a good ART and cryopreservation outcomes could be achieved by using human spermatozoa preserved in a liquid state in a TEST-Yolk-based buffer such as the Refrigeration Medium for at least 24 h post-ejaculation.

### 8.2. Citrate TEST-Yolk Buffer (CYB)

Whereas the use of the TEST-Yolk buffer achieved promising results in liquid storage of human spermatozoa regarding delayed ART or cryopreservation, it has not yet been reported to be applied in delayed clinical laboratorial testing of semen samples. In order to provide a solution for this, a human sperm preservation medium named as CYB was invented by R.J Aitken at the end of the last century [22]. The CYB medium consists of the following substances: TES/Tris and sodium citrate as basic buffer, fructose as the energy source, fresh egg yolk as the membrane protectant, and penicillin and streptomycin as antibacterial agents. Moreover, the addition of antioxidants such as α-tocopherol (vitamin E), catalase, and glutathione as free radical scavengers is recommended in medium. When the medium was supplemented with pentoxifylline, the motility could be further increased. The use of CYB medium is relatively simple. Fresh liquified human semen samples are diluted at a 1:1 ratio with the CYB medium. With the aid of the CYB medium, all aspects of semen functional competences could be kept unchanged, meeting the requirements of various andrological diagnostic tests after transportation for 25–30 h at ambient temperature. These tests include semen viability and motility, morphology, penetration of a cervical mucus substitute, acrosome reaction, and sperm–oocyte fusion. For instance, in a field trail, the sperm motility parameters were decreased significantly in seminal fluid but were maintained well in CYB medium before and after storage and transportation [23]. The CYB medium has shown many advantages compared with conventional the TEST-Yolk buffer. The compositions in CYB medium are similar to the TEST-Yolk buffer, and the addition of citrate and antioxidant offer spermatozoa better protection against unfavorable conditions. Moreover, preservation at ambient temperature could avoid cold shock during chilling, which may lead to irreversible injuries of spermatozoa [80]. Although original sperm characteristics in asthenozoospermic patients are preserved well using CYB medium, the effects of spermatozoa from oligozoospermic patients need to be verified. To the author’s best knowledge, CYB are not yet clinically available. Nevertheless, CYB medium offers considerable promise as a medium for the liquid storage of human semen.

### 8.3. Electrolyte-Free Solution (EFM)

As pointed out before, the reduced activity of sodium-potassium-dependent ATPase (Na^+^/K^+^ ATPase) that is sensitive to ATP depletion and hypothermia is considered to hamper the prolonged storage of the sperm. Inhibition of Na^+^/K^+^ ATPase leads to the influx of extracellular sodium ions, resulting in cellular injury. Several studies have revealed the harmful effects on sperm motility when the sodium and potassium ions are presented in the liquid storage medium. Based on the above observations, a method for the long-term preservation of human spermatozoa in an Electrolyte-free Medium (EFM) that removed both sodium and potassium ions at 4 °C was developed [59,266]. In this method, fresh semen samples were firstly centrifuged in an electrolyte-free multiple layers Percoll gradient, then the pelleted sperm were preserved in a EFM that consisted of 0.33 M glucose solution with 3% BSA immediately at 4 °C. Before using spermatozoa preserved in EFM, Ham’s F-10 medium was added and incubated for 1 h to induce reinitiation. Results showed that preserving sperm in EFM at 4 °C could maintain motility of sperm from both normal and asthenozoospermic semen samples for at least weeks, much better than solutions containing sodium or potassium and TEST-yolk buffer. Follow-up studies showed that the morphology, viability, ATP concentration, and acrosome status of sperm were also conserved in EFM cold preservation [124,267]. Besides, the chromatin integrity of sperm preserved using this method after one week was maintained better than cryopreservation and were proven to be fully functional in ICSI [11,34]. EFM was initially developed for the ART, the same as the Refrigeration Medium. Storage of human spermatozoa in cold EFM aims to provide an alternative to cryopreservation when men can provide semen several days before the ART, which is simpler and could avoid cryodamage. Both the EFM and the Refrigeration Medium have pros and cons. EFM is able to preserve spermatozoa for a much longer time than the Refrigeration Medium, maintaining the fertility capacity for up to several weeks versus 4 days at 4 °C. However, compared with the broad scope of applications of the Refrigeration Medium, preserving sperm in EFM requires the removal of semen plasma by gradient centrifugation, limiting its use in transportation of semen samples and excluded it from applications in laboratory testing. In addition, the cryopreservation of spermatozoa after EFM cold storage has not been reported yet. Indeed, EFM significantly extends the storage duration of human sperm in the liquid state and represents an attempt to develop a roadmap for guiding the design of novel liquid storage methods.

### 8.4. Artificial Seminal Fluid

Although the above-mentioned extenders, such as CYB or EFM, played a good protective role in maintaining sperm parameters or fertility quality, their compositions are much different from the real human seminal fluid. It is interesting to develop a freezing-free preservation medium to simulate the physical and chemical properties of human semen. A semen simulant, called “artificial seminal fluid”, was reported by Owen et al, which was based upon an extensive review of the literature on properties and constituents of human semen [56]. The considerations were focused on pH and buffering capacity, osmolarity, viscosity, and the main chemical components such as citrate and protein. Their reference values and concentrations were summarized, and the formulation of this semen simulant was described in detail. The artificial seminal fluid was originally used to support research in reproductive health, especially in drug release kinetics and delivery vehicle distribution, as a fluid representative of those that will be encountered within the vagina. So, a more simplified and stable seminal fluid simulant was designed and characterized, which is necessary for a variety of in vitro product evaluations [268]. Despite that, the author noted that the artificial seminal fluid was not developed as a culture medium for spermatozoa; its promising potential in sperm storage was soon discovered and was used in sperm cryopreservation. After slight modifications, the artificial seminal fluid was used in vitrification of human spermatozoa without the cryoprotectant. Compared with the vitrification using the human tubal fluid and the normal human seminal fluid, vitrification using the artificial seminal fluid had significantly higher sperm viability and motility [269]. Subsequently, the impact on sperm parameters and DNA status of asthenozoospermic ejaculates preserved in the artificial seminal fluid were investigated. The asthenozoospermic ejaculates were firstly processed with density gradient centrifugation, and then the pellet was divided into four aliquots and resuspended with the artificial seminal fluid, Ham’s F10 medium, normozoospermic seminal fluid, and asthenozoospermic seminal fluid, respectively. Sperm parameters and DNA integrity were assessed after incubation for up to 24 h. Better preservation of sperm motility was observed in the artificial seminal fluid than the other three medium fluids after 24 h. In addition, regarding the DNA fragmentation index, the artificial seminal fluid was similar to normozoospermic seminal fluid but was significantly lower than the Ham’s F10 medium and the asthenozoospermic seminal fluid [119]. The results showed that the artificial seminal fluid had the potential to be applied in sperm selection. The results also shed light on its potential for liquid storage of human sperm, although this finding is not the aim of the current study. It is interesting to further explore the effect of preservation using artificial seminal fluid, such as the simple addition to it in normal human semen at 1:1 volume ratio.

## 9. Future Perspective

Despite the developments of extenders, prolonged storage of spermatozoa inevitably compromises the sperm quality and requires further improvement. Moreover, the underlying mechanism of injuries during this procedure remains inclusive, which may provide crucial information for improving the preservation effect. Recently, there have been many encouraging outlooks and advanced technical attempts that have been made to promote the liquid storage, among which nanotechnology and omics technology are most promising and show significant advantages [270,271,272]. Although these novel techniques in sperm storage are mostly used in animal sperm cryopreservation, they have demonstrated beneficial results on liquid storage of animal experiments, and we are confident that they will also provide great inspirations in liquid storage of human spermatozoa.

### 9.1. Nanotechnology and Drug Delivery System

In conventional extenders, protective substances are added into extenders directly that may make them less effective or bring side effects. With the development of nanotechnology and drug delivery technology, the use of nano-sized protective additives or nanovesicles for carrying these protective additives has shown superiority in the field of semen preservation.

Nanotechnology, which is very promising for numerous biomedical applications, has been introduced in sperm preservation in recent years. Some metal nanoparticles such as zinc oxide, sliver, and selenium nanoparticles serve as efficient additives in extenders due to their outstanding antioxidative or antibacterial properties. Compared with vitamin C, E, and corresponding inorganic trace elements, nano-sized particles of zinc and selenium showed more advantageous effects on camel epididymal spermatozoa cooled at 5 °C for 2 h through the increase of bioavailability and alleviation of toxicity [273]. However, potential risks linked with these nanomaterials should not be overlooked. Metal oxide nanoparticles have been reported to be detrimental to sperm quality in many in vitro studies, as the result of triggering excessive ROS production [274,275]. Human spermatozoa incubated with zinc oxide nanoparticles for no more than 3 h decreased sperm viability, suggesting that the role of nanoparticles in extenders might be significantly different between cryopreservation and liquid storage. Moreover, the effects of residual nanoparticles in spermatozoa, such as possible endocytosis, require further research [276]. Except for inorganic metal elements, some organic substances play protective roles in sperm preservation and can be prepared as nanoformulations that overcome their limitations such as low stability and hydrophobicity. A vitamin E delivery system using nanoemuisions carriers showed significantly better sperm preservation effects compared with free vitamin E [277]. Nanosized curcumin extracts, lecithin can also be added into extenders to improve the cryosurvival of animal sperm [278,279]. Furthermore, the addition of vitamin E, combined with selenium nanoparticles, improved the post-thawing quality and oxidative variables of rooster semen [280]. Until now, although few applications are available about nanoparticles used in liquid storage of sperm preservation, their potential in this field has gained more and more interest.

As suitable carriers in drug delivery systems due to their excellent biocompatibility and biodegradability, liposome vesicles have been used in sperm preservation in recent years. Liposome was proven to be a better carrier-compound than DMSO for organic solutes promoting freezing tolerance of spermatozoa [281]. Fat soluble antioxidants encapsulated in liposomes might increase water solubility and stabilize the temperature and pH of antioxidants. For example, liposome-encapsulated lycopene and quercetin showing stronger antioxidative abilities results in the improvement of the rooster sperm quality [282]. Besides, liposome is also an appropriate carrier for membrane protectants. Addition of liposome encapsulated soy lecithin and cholesterol was proven to have a higher post-thaw viability and motility of human spermatozoa in comparison with egg yolk [240]. The components of liposome such as fatty acids and phospholipids can be used as ingredients to repair the damaged plasma membrane [283]. Liposome-based extenders are more effective than egg yolk and soy lecithin-based extenders. Except for liposomes, extracellular vehicles (EVs), such as exosomes, contain proteins, lipids, and nucleic acids that have important functions in male reproduction, modulating sperm functions, and male fertility [284]. Recent studies have found these EVs may play an important role in sperm preservation. Seminal plasma-derived and stem cell-derived exosomes were reported to be beneficial to sperm cryopreservation or stored in the liquid state [285,286]. Moreover, alginic acid microencapsulation of human sperm is shown to significantly improve the cumulative membrane integrity and DNA fragmentation following storage for 24 h at 37 °C [287].

### 9.2. Omics Technologies

The inter-male variation of sperm cryo-survivability and cryo-tolerance was associated with the difference in spermatozoa and the seminal plasma, while their detailed molecular mechanism has not been methodically explained [288,289]. It has been proved that some genes, proteins, and metabolites are correlated with freezability, allowing the development of novel additives to improve the effect of freezing medium [216]. In recent years, advanced omics technologies, such as transcriptomics, proteomics, and metabolomics, have emerged as powerful tools to identify specific freezability biomarkers and to explore key molecules involved in the cryoinjuries. These omics technologies also show potential in improvement of sperm liquid preservation processes and have begun to be applied in domestic animal species.

Cryopreservation is able to affect transcriptomics profiling of spermatozoa, including differential expression of genes that helps to resist or repair cryoinjuries. For example, the upregulation of glutamate-cysteine ligase catalytic subunit (GCLC) gene was observed to take part in the response of sperm to cold shock and oxidative stress during the freezing and thawing cycle [290]. Except for transcriptomics, different protein profiles of spermatozoa and seminal plasma are probably associated with sperm preservation ability [291]. Sperm cells possess a higher level of proteins and enzymes, which guard the sperm against oxidative stresses such as voltage-dependent anion channel 2 and heat shock protein 90, which have been identified to be associated with better sperm preservation ability [292]. Wang et al. identified that the GSK3A substrate network, which may be a key kinase participating in sperm motility, was abnormally activated in cryodamage of sperm though analysis of more specific phosphoproteome [293]. The maintenance of sperm motility after preservation for 24 h at 15 °C diluted with milk is linked with differences in the seminal plasma proteome, and several sperm membrane proteins and capacitation-related proteins may play a role in the effect of liquid storage [294]. Furthermore, the metabolite profiles of sperm and seminal plasma determined by metabolomic approaches, such as nuclear magnetic resonance spectroscopy, can characterize some key end-products related to freezing resistance [295]. The seminal plasma metabolome between high and low freezability ejaculates of boar were revealed using UHPLC-qTOF-MS analysis. D-aspartic acid, N-acetyl-L-glutamate, and inosine served as candidates to assess sperm cryopreservation resistance [296]. Moreover, metabolite profiling of the pig sperm after storing for 72 h at 17 °C were identified and quantified though NMR analysis. Leucine, hypotaurine, carnitine, and isoleucine were found to be associated with the ability to withstand liquid storage, suggesting these molecules have the potential to predict the sperm resilience to liquid storage [297]. However, the putative biomarkers identified between cryopreservation and liquid storage of the same animal, showed obvious discrepancies. Nevertheless, these findings will pave the way for omics technologies applied in the liquid storage of human sperm.

## Figures and Tables

**Table 1:** Results of sperm parameters reveals effects of different conditions during prolonged liquid storage of human spermatozoa.

Parameters/Assays	Extender/Pre-treatment	Storage Temperature	Storage Duration	Main Results	Reference
Viability,	SP	4, 20 or 37 °C	18 h	Semen should be kept at 20 °C if any delay happens	[105]
Motility		4, 25 or 37 °C	1 h	Semen should be stored at 37 °C after collection	[88]
and motion parameters	TEST-yolk buffer	5 °C	96 h	50% motility recovery after 96 h	[21]
		2–5 °C	48 h	70% motility recovery after 96 h	[110]
		4 °C	72 h	52% viability and 14% PR recovery after 72 h	[106]
		24, 37 or 40 °C	24 h	Motility recovery was best preserved at 24 °C	[45]
	Human follicular fluid, PBS	37 °C	12 h	30% and nearly 0% motility and PR recovery after 12 h in human follicular fluid and in PBS	[111]
	Modified Tyrode’s solution	3 or 37 °C	24 h	50% and 1% motility recovery after 24 h at 3 °C and 37 °C	[112]
		4, 22 or 37 °C	5 days	Viability and Motility recovery (85% and 50%) was best preserved at 22 °C after 5 days	[113]
		4, 22 or 37 °C	5 days	Viability and Motility recovery (80% and 50%) was best preserved at 22 °C after 5 days	[114]
	Citrate TEST-yolk buffer	RT	25–30 h	Motility recovery are nearly 100% after 24 h at RT, VCL, VAP and PR are sustained	[22]
	EFM	4 °C	Record till 0	Viability and motility maintained for up to 42 and 28 days	[11]
	Modified Human tubal fluid	4, 23 or 37 °C	Record till 0	>50% Viability and Motility recovery was best preserved at 22 °C after 21 and 10 days	[115]
		4 °C	Record till 0	Viability and motility maintained <2 weeks and <4 weeks	[11]
		24 °C	7 days	Viability and Motility recovery (53% and 10%) after 5 days	[37]
		24, 37 or 40 °C	24 h	Motility recovery was best preserved at 24 °C	[45]
	Modified Ham’s F-10 media	37 °C	24 h	Viability and Motility recovery (62% and 1%) after 24 h	[116]
		RT, 37 °C	72 h	Testicular sperm motility was optimized at RT after 24 h of incubation	[14]
		4–6 or 25 °C	12 days	Motility recovery (8% at 4–6 °C and 2% at 25 °C) after 7 days	[108]
		RT, 37 °C	4 h	Viability and Motility recovery (73% and 67%) of asthenoteratozoospermia and no difference between RT and 37 °C	[117]
	PBS/Human follicular fluids	37 °C	12 h	Sperm motility and VCL were significantly stimulated	[111]
	SP, G-IVF^TM^	RT, 37 °C	24 h	25% motility recovery at RT with no treatment. Motility was totally lost at 37 °C in SP or G- IVF^TM^	[118]
	SP, Ham’s F10 media, ASF	RT	24 h	Viability and Motility in Ham’s F10 and ASF are comparable and better than no SP	[119]
	PBS plus glucose	4, 22 and 37 °C	10 days	Motility recovery was 20% at 22 °C	[120]
	BWW medium	37 °C	3 days	Motility and PR recovery was 12% and 0% at 37 °C, significant increase in VSL, VCL, and VAP after penicillamine supplementation	[109]
	Earle’s balanced salt solution	RT, 35 °C	24 h	Viability and Motility recovery (95% and 90%) was best at RT	[121]
	BM1	RT, 35 °C	24 h	Motility recovery was 76% at RT	[122]
	G-IVF^TM^	4–6 or 25 °C	12 days	Motility recovery was 8% at 4–6 °C and 1.5% and 25 °C after 7 days	[108]
Morphology	Ringer Glucose Phosphate buffer	21 or 37 °C	22 h	Prolonged (>2 h) sperm manipulations for ART should be performed at 21 °C rather than 37 °C	[40]
	Modified Human tubal fluid	24 °C	7 days	Deterioration in morphology is slower during 7-day incubation	[37]
	BM1	RT, 35 °C	24 h	Morphologically normal spermatozoa were significantly higher in RT compared with 35 °C	[122]
	Not clear	37 °C	5 h	Morphologically normal spermatozoa increased after incubation	[103]
Hamster egg	TEST-yolk buffer	2–5 °C	48 h	Increased significantly following storage	[110]
fusion	Citrate TEST-yolk buffer	RT	25–30 h	Remained unchanged	[22]
Acrosome					
Function					
FITC-PNA stained	Citrate TEST-yolk buffer	RT	25–30 h	Remained unchanged	[22]
	Ham’s F10 medium	37 °C	22 h	No effect	[5]
Oxdative Stress					
ROS level	Ham’s F10 medium	37 °C	22 h	Significant increase after 22 h	[5]
	SP	4, 25 or 37 °C	1 h	Semen should be stored at 37 °C after collection	[88]
Membrane damage					
MMP(JC-1)	SP	Not clear	4 h	Worse MMP is associated with a significant decline in motility over time	[123]
HOS	Modified human tubal fluid	24 °C	7 days	Membrane integrity dropped down to 80%	[37]
	EFM	4 °C	4 weeks	Membrane integrity were significantly better than TEST-yolk buffer	[124]
SYBR14/PI and JC-1	PBS plus glucose	4, 22 and 37 °C	10 days	Viable sperm with functional mitochondria are >40% after 10 days	[120]
SYBR14/PI	EFM	4 °C	Record till 0	Sperm with intact cell membrane maintained for up to 7 weeks	[34]
Annexin V/PI	G-IVF^TM^	4–6 or 25 °C	12 days	The mean percentage of late apoptotic sperm cells increased significantly from day 0 to day 12	[107]
	Human tubal fluid	37 °C	24 h	The mean percentage of apoptotic sperm cells increase nearly 50% after 24 h	[125]
LPO					
TBA assay	Citrate TEST-yolk buffer	RT	25–30 h	Preserve motility but not suppress lipid peroxidation	[22]
	Ham’s F10 medium	37 °C	22 h	Significant increase after 22 h	[5]
DNA damage					
	EFM	4 °C	2 weeks	SDF was slightly higher after than before storage	[10]
SCSA	SP, G-IVF^TM^	RT, 37 °C	24 h	SDF increased, but at RT was significantly lower than 37 °C	[118]
SCD	SP, human tubal fluid	On ice, RT	24 h	RT for 24 h significantly increased SDF	[126]
	SP, PBS	37 °C	24 h	SP increase SDF after 2 h so that it must be removed	[127]
	Ham’s F10 media	37 °C	3 h	SDF was significantly higher after 2 h	[13]
		37 °C	22 h	SDF was significant increased after 22 h	[5]
	Human follicular fluid	37 °C	24 h	Co-incubation with HFF could reduce SDF	[128]
	SP, Ham’s F10 media, ASF	RT	24 h	SDF preserved in ASF are similar with normal SP	[119]
TUNEL	BM1	RT, 35 °C	24 h	Increase of SDF was significantly lower at RT than at 35 °C	[122]
	Modified Tyrode’s medium	37 °C	3 days	Results in significant DNA damage	[114]
Comat assay	EFM	4 °C	1 week	DNA integrity was significantly higher than cryopreservation	[34]
Acridine orange	G-IVF^TM^	4–6 or 25 °C	12 days	Sperm DNA remained unchanged after 12 days at both temperatures	[107]

Except for no treatment (left in seminal plasma), diluted with TEST-yolk buffer, and citrate TEST-yolk buffer, spermatozoa incubated in other mediums were all prepared by washing, the swim-up method, or density gradient centrifugation. Abbreviation: SP: Seminal Plasma; PR, Progressive Motility; PBS, Phosphate Buffer Solution; RT, Room Temperature; EFM, Electrolyte-free Medium; ASF, Artificial Seminal Fluid; BWW, Biggers, Whitter and Whittingham; VCL, curvilinear velocity; VSL, straight-line velocity; VAP, average path velocity; PNA: Arachis hypogea (peanut) agglutinin; ART, Assisted Reproductive Technology; HOS, Hypo-osmotic Swelling; ROS, Reactive Oxygen Species; MMP, Mitochondrial Membrane Potential; LPO, lipid peroxidation; TBA, Thiobarbituric Acid; SDF, Sperm DNA Fragmentation; SCSA, Sperm Chromatin Structure Assay; SCD, Sperm Chromatin Dispersion Assay; HFF, Human follicular fluid; TUNEL, Terminal Transferase-mediated DNA end-labeling method. Cultural Medium: BM1: sperm preparation medium (EUROBIO.TA 10.07.59 France); G-IVF^TM^: Vitrolife Inc., Sweden.

**Table 2:** Pregnancy outcomes of extended culture of human spermatozoa in an assisted reproductive technology procedure.

ART	Time Interval of Incubation	Extender/Pre-Treatment	Temperature	Main Results	Reference
ICSI	3.66 ± 2.26 h	Swim-up/Commercial Culture Medium	37 °C	No adverse effects on the pregnancy and live birth rates	[12]
IVF/ICSI	Home collection to processing: within 1.5 h	DGC/Commercial Culture Medium	Close to 37 °C	No significant difference in oocytes, embryos, pregnancy rates	[9]
ICSI	2 weeks	Electrolyte-free medium (EFM)	4 °C	High pregnancy rate and high physical status scores in the resulting newborns	[10]
IVF/ICSI	Home collection to processing: up to 2 h	DGC/HTF	37 °C	Does not negatively impact sperm parameters, fertilization rate, pregnancy rates	[8]
ICSI	5 h	DGC/Commercial Culture Medium	37 °C	No influence on fertilization rates	[103]
IUI	Within 24 h	DGC/Commercial Culture Medium	21–37 °C	No negative effect on pregnancy rate	[149]
IUI	Not clear	DGC/Commercial Culture Medium	RT or 37 °C	No negative effect on clinical pregnancy rate when preserving at RT or at 37 °C	[148]
IUI	Semen collection at clinic or at home. Delaying semen processing: 0.5 h up to 1 h; Delaying IUI: 1.5 h up to 2 h	DGC/Commercial Culture Medium	21–40 °C or 37 °C	Delaying semen collection, processing and IUI compromise the pregnancy outcome	[151]
IUI	40–80 min	DGC or Swim-up/Commercial Culture Medium	RT or 37 °C	Sperm preparation for 40–80 min has a potential positive effect on pregnancy rate.	[152]
IUI	107 min between semen production and IUI	DGC/HTF	Not clear	Time intervals between semen production and analysis and IUI significantly influenced clinical pregnancies and live births and should be kept low.	[153]
IUI	Semen collection to washing: 40 min Semen washing to IUI: 60 min Semen collection to IUI: 90 min	Swim-up/Commercial Culture Medium	RT	Shorter time interval between semen collection, washing and IUI resulted in higher pregnancy rate.	[150]
IUI	24 h	TEST-Yolk Buffer/Commercial Culture Medium	5 °C	Similar pregnancy rates were obtained between the immediately IUI group and 24 h storage group	[51]
IUI	2 h	Swim-up/TEST-Yolk Buffer/Earle’s medium	4 °C	TYB significantly increased pregnancy rate in patients with unexplained infertility but not in those with male factor	[154]
IVF	2 h	Double-step washing technique/TEST-Yolk Buffer/Ham’s F-10 medium	RT	Significantly improved fertilization in couples who had low fertilization rates	[155]
IVF	2 h	DGC/TEST-Yolk Buffer/Commercial Culture Medium	RT	Incubation of TEST-Yolk Buffer produces significantly higher fertilization rates than standard IVF preparation	[156]
IVF	24 h	TEST-Yolk Buffer-treated: DGC/Ham’s F10 medium Fresh semen: swim-up/Ham’s F10 medium	4 °C	TEST-yolk buffer pretreatment of sperm for 24 h results in higher fertilization rates during IVF among suspected male factor patients.	[157]
IUI	Not clear	TEST-Yolk Buffer/BWW	Not clear	Higher pregnancy rates with TEST-Yolk than BWW	[158]
IVF	2 h	Percoll processed/TEST-Yolk Buffer/Milk/Ham’s F10 medium	5 °C	Preincubation of spermatozoa in milk or TEST-Yolk yields a similar IVF outcome	[159]

Abbreviation: RT, Room Temperature; BWW, Biggers, Whitter and Whittingham; ART, Assisted Reproductive Technology; DGC: Density Gradient Centrifugation; IVF: In Vitro Fertilization; ICSI: Intracytoplasmic Sperm Injection; IUI: Intrauterine Insemination; HTF: Human Tubal Fluid.

**Table 3:** The current typical extenders for the liquid storage of human sperm.

Names	RM	CYB	EFM	Artificial Seminal Fluid	Human Tubal Fluid
Components					
Buffers	Tris (80 mM)	Tris (46 mM)	-	NaCl (2.69 g/L); KCl (0.43 g/L); K_2_HPO_4_ (1.91 g/L); MgSO_4_ (0.54 g/L);	NaCl (5.93 g/L); KCl (0.35 g/L); KH_2_PO_4_ (0.05 g/L);
	TES (176 mM)	TES (108 mM)	-	ZnSO_4_·7H_2_O (0.5 g/L); CaCl_2_·7H_2_O (0.73 g/L); NaHCO_3_ (2.1 g/L); Sodium	NaCl (5.93 g/L); KCl (0.35 g/L); KH_2_PO_4_ (0.05 g/L);
		Sodium citrate (131 mM)	-	Citrate·2H_2_O (8.13 g/L); Sodium Pyruvate (0.374 g/L); Sodium Lactate (0.779 g/L)	CaCl_2_ ·2H_2_O (0.3 g/L); MgSO_4_·7H_2_O (0.05 g/L); Pyruvate (0.036 g/L); Lactate (4 g/L)
pH	7.0	7.3–7.5	7.0	7.4–7.7	7.4
Osmolarity (mOs/kg))	325 ± 2	290–320		325–354	280–295
Energy sources	Glucose (9 mM)	Fructose (95 mM)	Glucose (0.33 M)	Glucose·1H_2_O (1.12 g/L) and fructose (2.72 g/L)	Glucose (0.5 g/L)
Membrane protectant (*v*/*v*)	Egg yolk (20%)	Egg yolk (20%)	BSA (3%)	BSA (5.04 g/100 mL)	HSA (optional)
Antioxidants	-	α-tocopherol (1 mM)	-	Urate (0.07 g/L); urea (0.72 g/L)	
Antibiotics	Gentamicin (10 mg/L)	P-S (10000 U)	-	Gentamycin (40 mg/L)	P-S (10000 U)
Pre-treatment	1:1 dilution	1:1 dilution	Removal of SP	Removal of SP	Removal of SP
Storage Temperature	RT, 2–5 °C	RT	2–5 °C	RT	RT, 2–5 °C, 37 °C
Storage Duration	Up to 4 days	25–30 h	Up to 7 weeks	24 h	2–24 h (most of experiments)
Application	ART; Cryopreservation	Laboratorial test	ART	Pharmacy; ART	ART; Cryopreservation
Commercialization	Yes	No	No	No	Yes
Reference	[21,106]	[22,23]	[10,11,266]	[56,119]	[37,115,201]

Abbreviations: RM, Refrigeration Medium; CYB, Citrate TEST-Yolk Buffer; EFM, Electrolyte-free Medium; BSA, Bovine Serum Albumin; HSA, Human Serum Albumin; P-S, Penicillin and Streptomycin; SP, Seminal Plasma; RT, Room Temperature; ART, Assisted Reproductive Technology.
